# Structural characterization of *Myxococcus xanthus* MglC, a component of the polarity control system, and its interactions with its paralog MglB

**DOI:** 10.1016/j.jbc.2021.100308

**Published:** 2021-01-22

**Authors:** Srajan Kapoor, Akriti Kodesia, Nidhi Kalidas, Ashish, Krishan Gopal Thakur

**Affiliations:** 1 Structural Biology Laboratory, Council of Scientific and Industrial Research-Institute of Microbial Technology, G. N. Ramachandran Protein Centre, Chandigarh, India; 2 Council of Scientific and Industrial Research-Institute of Microbial Technology, G. N. Ramachandran Protein Centre, Chandigarh, India

**Keywords:** *Myxococcus xanthus*, MglC, MglB, protein–protein interactions, RLC7 family, isothermal calorimetry, SAXS, bacterial motility, cell polarity, polarity reversal, D_max_, maximum linear dimension, ITC, isothermal titration calorimetry, LAMTOR2, late endosomal/lysosomal adaptor and MAPK and MTOR activator 2, MglA, Mutual gliding-motility protein A, MglB, Mutual gliding-motility protein B, MglC, Mutual gliding motility protein C, MSA, multiple sequence alignment, NSD, normalized spatial discrepancy, R_g_, radius of gyration, RLC7, Regulatory Light Chain 7, RomRX, Required for motility response regulator complex, SAXS, small-angle X-ray scattering, Se-SAD, Selenomethionine Single-wavelength anomalous diffraction, SEC, size-exclusion chromatography, TEV, tobacco etch virus

## Abstract

The δ-proteobacteria *Myxococcus xanthus* displays social (S) and adventurous (A) motilities, which require pole-to-pole reversal of the motility regulator proteins. Mutual gliding motility protein C (MglC), a paralog of GTPase-activating protein Mutual gliding motility protein B (MglB), is a member of the polarity module involved in regulating motility. However, little is known about the structure and function of MglC. Here, we determined ∼1.85 Å resolution crystal structure of MglC using Selenomethionine Single-wavelength anomalous diffraction. The crystal structure revealed that, despite sharing <9% sequence identity, both MglB and MglC adopt a Regulatory Light Chain 7 family fold. However, MglC has a distinct ∼30° to 40° shift in the orientation of the functionally important α2 helix compared with other structural homologs. Using isothermal titration calorimetry and size-exclusion chromatography, we show that MglC binds MglB in 2:4 stoichiometry with submicromolar range dissociation constant. Using small-angle X-ray scattering and molecular docking studies, we show that the MglBC complex consists of a MglC homodimer sandwiched between two homodimers of MglB. A combination of size-exclusion chromatography and site-directed mutagenesis studies confirmed the MglBC interacting interface obtained by molecular docking studies. Finally, we show that the C-terminal region of MglB, crucial for binding its established partner MglA, is not required for binding MglC. These studies suggest that the MglB uses distinct interfaces to bind MglA and MglC. Based on these data, we propose a model suggesting a new role for MglC in polarity reversal in *M. xanthus*.

*Myxococcus xanthus* is an anaerobic, rod-shaped, gram-negative δ-proteobacteria. It is widely studied for its complex social behavior, life cycle, and motility (1, 2). It exhibits two types of motilities, S “social” motility and A “adventurous” motility (3, 4). In the S-motility, a large group of bacterial cells coordinate to move together. The S-motility uses the type IVa pilus filaments that are formed at the leading pole and moves the cells forward (5), whereas in the A-motility, single cells move at the periphery of bacterial colonies to explore the surrounding environment (6). The A-motility is type IVa pilus independent and uses Agl-Glt motility machinery that assembles at the leading pole and provides directionality (7). The common feature of both types of motility systems is the leading pole assembly of type IVa pili and Agl–Glt complex and their polar inversion by 180° at the opposite poles (8, 9, 10). Regulation of pole reversal is essential for modulating motility, which aids adaption and survival in *M. xanthus* (11). The polarity reversals are controlled by “frizzy” signal transduction proteins that act as a “switch control system” (12, 13, 14). The Frz system controls the “polarity control system”, that is, Mutual gliding-motility protein A (MglA), Mutual gliding-motility protein B (MglB), and Required for motility response regulator complex (RomRX) (15, 16, 17).

As shown in Figure 1, before the reversal, MglA, a GTPase, in its GTP-bound active form, is present at the leading pole (18, 19, 20), whereas MglB, a GTPase-activating protein, is present at the lagging pole along with RomRX that act as guanine exchange factor (21, 22, 23). The “frizzy” signal transduction proteins, part of the Frz system, are known to start and regulate the polarity reversal process (24, 25). The FrzE phosphorylates its response regulators FrzX and FrzZ known to interact and modulate MglB and MglA, respectively (26, 27). The exact sequence of events for the reversal process is not known. However, the MglA proteins first dissociate from the leading pole because of FrzZ signal and travel toward the lagging pole (8). MglA and MglB colocalize at the leading pole for about 30 s, and during this time, the GTPase activity of MglB is not functional because of inactivation by FrzX (17). Then the MglB proteins detach from the lagging pole, move to the opposite pole, and RomRX loads more MglA–GTP molecules to the new leading pole. The RomRX complex then slowly dissociates from the new leading pole and moves to the opposite pole, marking the formation of the new leading and lagging poles (8). The time taken by RomRX to detach from one pole and get accumulated at the opposite pole in a sufficient amount marks the refractory period as no reversal activity of MglA and MglB can occur at this time (28).

**Figure 1:**
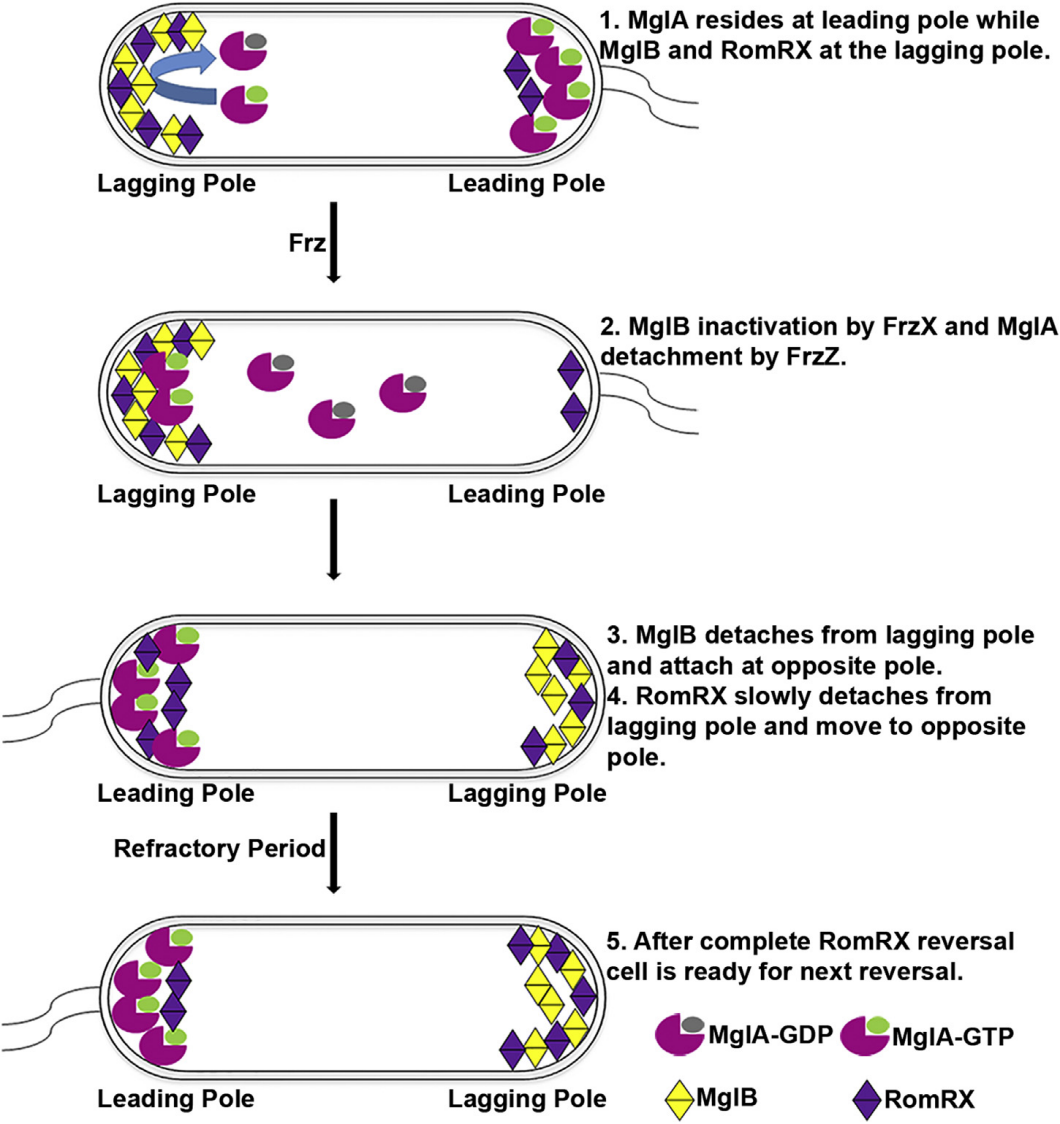
**Regulation of polarity reversal in *Myxococcus xanthus*.** Polarity reversals of MglA, MglB, and RomRX *via* Frz signaling controls the A motility (gliding motility, Agl–Glt complex) and S motility (swarming motility, type IV pili) of *M. xanthus*. MglA, Mutual gliding-motility protein A; MglB, Mutual gliding-motility protein B; RomRX, Required for motility response regulator complex.

Currently, the mechanism of the detachment of MglB and RomRX proteins from pole and their attachment to opposite poles is not clear. Recently, a new protein, Mutual gliding motility protein C (MglC), has been identified as a member of the Regulatory Light Chain 7 (RLC7) family protein, probably formed by gene duplication and divergence, that over time has lost its ability to bind MglA (29). MglC is required for polarity reversal and interacts with MglB and RomR. MglC is recruited asymmetrically at the lagging poles by RomR as in the absence of RomR, the MglC protein is diffused in the cytoplasm (29). MglC is localized in a bipolar manner in the presence of RomR, but the presence of MglB mediates localization of MglC mainly at the lagging pole. Using bacterial two-hybrid system, MglC has been shown to interact with MglB and RomR (29). Upon deletion of MglC, *M. xanthus* cells remain motile but the rate of cellar reversals for motility apparatus reduces significantly, suggesting MglC is probably involved in regulating the rate of cellular reversals for MglB and RomR (29). To understand the role of MglC in regulating pole reversals and functional divergence from its paralog MglB, it is important to structurally characterize MglC and its interactions with the binding partners.

Here, we determined the crystal structure of MglC (selenomethionine derivative) and MglC (native) at 1.85 Å and 2.19 Å resolution, which revealed structural similarity with MglB despite sharing poor sequence conservation. Comparative structural analysis also revealed distinct structural features compared with other RLC7 family proteins. We further established and characterized MglB and MglC protein–protein interactions using analytical size-exclusion chromatography (SEC) and isothermal titration calorimetry (ITC). Based on data from site-directed mutagenesis, small-angle X-ray scattering (SAXS), SEC, ITC, and protein–protein docking studies, we propose a structural model of MglBC protein–protein complex.

## Results

### Multiple sequence alignment of MglC and MglB reveals distinct sequence features

Based on the multiple sequence alignment (MSA) and predicted secondary structure assignments, MglC has been predicted to be a member of RLC7 family proteins (30). Because MglB is also a member of this family sharing ∼8% sequence identity and ∼17% similarity with MglC, we performed MSA to identify the conserved regions among these proteins. Although both these proteins share poor sequence identity, as expected, we observed only few key highly conserved residues. The representative MSA of MglC and MglB with sequences sharing >30% identity is shown in Fig. S1. All the annotated MglC homologs are shorter than those of MglB because of the absence of the extra N- and C-terminal residues (Fig. S1, *A* and *B*). These extra N-terminal residues of MglB adopt a β-strand conformation, whereas the C-terminal residues adopt an α-helical conformation and linker residue connecting α3-α4. These extra N- and C-terminal residues are functionally important in mediating MglA–MglB interactions (20).

MSA also highlights various other key differences and similarities among MglC and MglB proteins (Fig. S1). As shown previously by McLoon *et al.*, (29) highly conserved G27 in MglC (structurally equivalent residue G38 in MglB) is crucial for the formation of turn connecting β1-β2 strands. We also noticed that G103 in MglC (structurally equivalent residue G112 in MglB) present in α3 is also highly conserved. The residue at 106 position in MglC (115 in MglB) is also occupied by positively charged amino acid in both MglC and MglB. In MglC, we also observed another conserved G67, which is absent in MglB. In MglC, the residue number 62 is occupied by negatively charged residues, whereas in MglB, this equivalent position 79 is occupied by positively charged residue. It has been previously shown that F25, D26, and I28 (FDI sequence motif) of MglC might be involved in the MglBC interactions (29). Our MSA analysis suggests that highly conserved I28 residue of MglC is absent in MglB. The MglC is characterized by the presence of negatively charged [D/E]26 but is not conserved in MglB. However, as also observed by McLoon *et al.*, (29) there is the presence of conserved D36 in MglB near this position.

### Crystal structure of MglC

**Figure 2:**
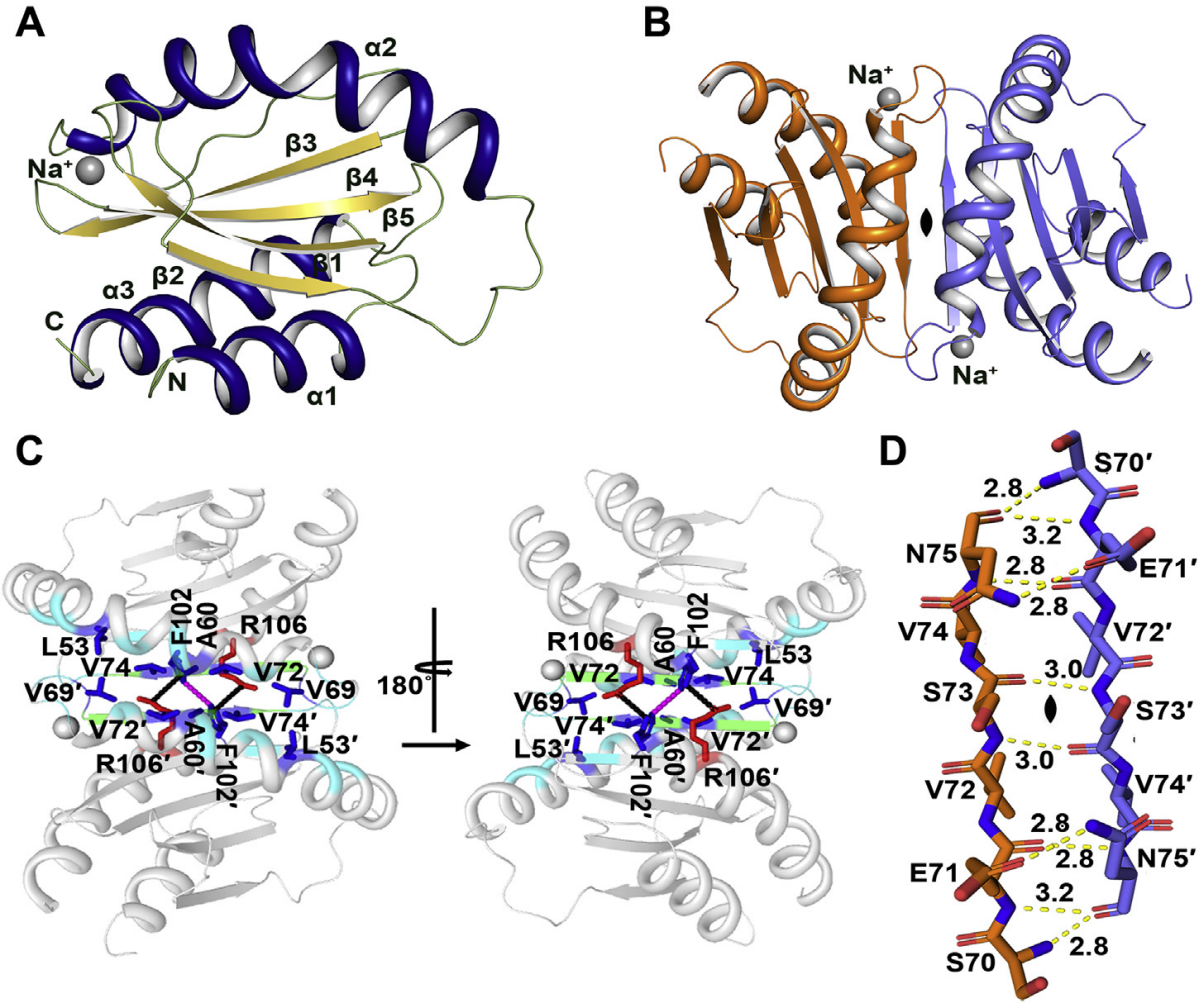
**Structural analysis of MglC.***A*, a cartoon representation of MglC monomer (asymmetric unit) colored according to the secondary structural elements (helices: *deep blue*, β-sheets: *yelloworange*, loops and turns: *smudge*). *B*, the biological assembly of MglC generated by applying twofold crystallographic symmetry. The protomers are shown in *orange* and *slate*. *C*, the detailed view of the dimeric interface. Residues involved in hydrophobic interactions are shown as *blue sticks*, R106 and F102 involved in cation-π interaction (*black dotted line*) are highlighted as *red* and *blue* sticks, respectively. The F102 from both protomers are involved in aromatic–aromatic interaction shown as *magenta dashed line*. The β sheet formed at the interface stabilized by main chain hydrogen bonds is shown in *green*. The remaining residues at the interface are shown in *cyan*. *D*, the hydrogen bonds formed by main chain and side chain interactions at the β sheet extension are shown as *yellowlines*. MglC, Mutual gliding motility protein C.

We successfully crystallized and determined the crystal structure of MglC. MglC crystallized in P6_5_22 space group (a = 96.69 Å, b = 96.69 Å, c = 58.28 Å, α = 90°, β = 90°, γ = 120°) with one molecule in an asymmetric unit. There was no good structural template to use as a model for solving structure using molecular replacement. The closest homologue available at RCSB PDB shared ∼8% sequence identity. So, we solved the crystal structure using Selenomethionine Single-wavelength anomalous diffraction (Se-SAD) experimental phasing technique at 1.85 Å resolution. The final refined model contains all 120 residues of MglC. The crystal structure revealed the typical RLC7 fold (α1β1β2α2β3α3) (Fig. 2*A*). We also solved the crystal structure of MglC in the native form at 2.192 Å resolution (a = 96.91 Å, b = 96.91 Å, c = 58.05 Å, α = 90°, β = 90° and γ = 90°) by molecular replacement using the structure obtained by Se-SAD as a template. The native and Se-SAD structures superpose well with r.m.s.d. of 0.144. MglC monomer contains five antiparallel β-strands sandwiched between three α-helices. MglC also contains a 3_10_ helix just after the α2 helix. The two β hairpins connect β1-β2 and β4-β5 strands. Other secondary structural elements include five β-bulges and eight β turns. We also observed an electron density for a metal ion in the crystal structure having a square pyramidal geometry. We placed Na^+^ which is coordinated by two water molecules and main chain oxygen atoms of L64, T66, and V69.

The structural analysis further suggests that, like other members of RLC7 family, MglC also forms homodimers and two protomers are related by crystallographic two-fold symmetry (Fig. 2*B*, Fig. S2*A*). PDBePISA (31) analysis suggests that MglC monomer has a total surface area of 6277 Å^2^ and the dimer interface buries 792 Å^2^ area (∼12.61%). The total surface area of MglC dimer is 10,970 Å^2^ with the 1580 Å^2^ (∼14.4%) being buried. The ΔG^int^ predicted using PDBePISA (31) for the dimer association is −9.1 kcal/mol and ΔG^diss^ is 3.9 kcal/mol, suggesting that MglC may form a stable dimer. MglC dimerization is mediated by β sheet extension, that is, β3 from each monomer comes together to form an antiparallel sheet consisting of ten β strands (five strands from each monomer) sandwiched by four helices on one side and two helices on the other side. The dimer is stabilized by six main chain hydrogen bonds and four hydrogen bonds involving side chains between the interacting β strands at the binding interface. Besides these, several nonbonded interactions are also observed between the interacting β strands and α2 helices at the binding interface (Fig. 2*C*, Fig. S2*B*). The residues involved in the formation of main chain H-bonds at the dimeric interface, that is, S70, E71, S73, V74, and N75 are highly conserved in MglC. Residue N75 and E71 also form hydrogen bonds with E71 and N75 of the other monomer (Fig. 2*D*, Fig. S2*B*). The α2 helices form hydrophobic contacts between chain A and chain B *via* the residues L53-V69′, A60-A60′, V69-L53′, V69-V74′, V72-V74′, V74-V69′, V74-V72′, and F102-F102′. The F102-F102 is also involved in aromatic–aromatic interactions. Besides this, F102 and R106 form a cation-п interaction (Fig. 2*C*, Fig. S2*B*). To further study the oligomeric status of MglC in the solution, we performed analytical SEC experiments. MglC eluted predominantly at ∼17 ml corresponding to a molecular weight of ∼35 kDa close to the expected size of the dimer (∼32 kDa). This further supports the crystallographic observations, hence confirming that MglC predominantly exists as a dimer in the solution (Fig. S2*A*).

**Figure 3:**
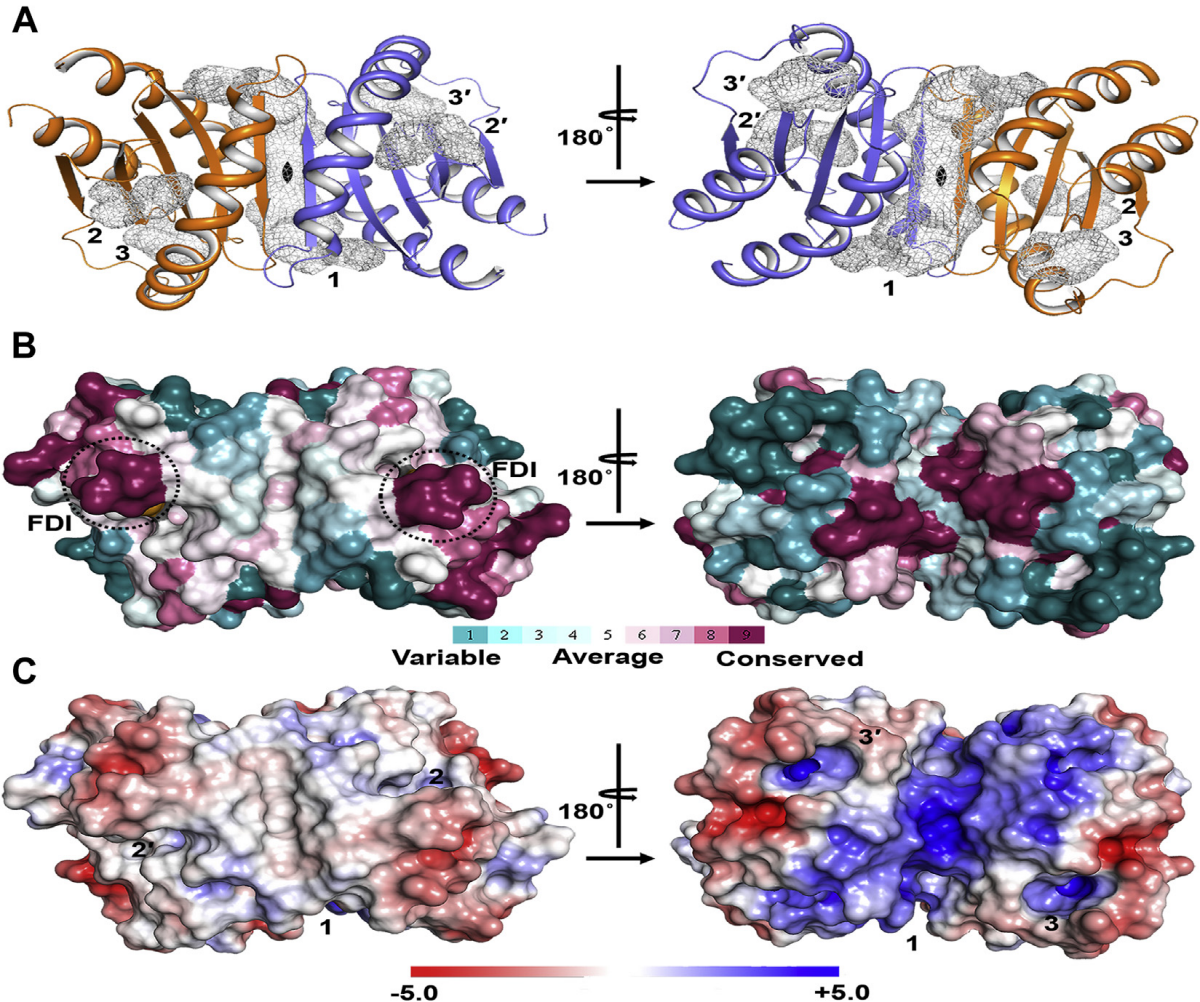
**Surface analysis of MglC.***A*, cleft analysis of MglC shows the presence of 3 clefts in MglC. Cleft 1 is the largest and is formed at the interface of dimer with the volume of ∼3946 Å^3^. This cleft mainly contains highly conserved positively charged residues. *B*, Consurf analysis (33, 34, 35, 36, 37) showing sequence conservation mapped on to the MglC crystal structure. The highly conserved region marked by *black dotted circle* includes the ‘FDI’ sequence motif previously shown to be involved in interacting with MglB. *C*, electrostatic surface potential of MglC showing distribution of negatively (*red*) and positively (*blue*) charged regions (38). MglB, Mutual gliding-motility protein B; MglC, Mutual gliding motility protein C.

To obtain information about the probable binding sites of MglC with MglB and RomR, we preformed cleft analysis of MglC using PDBsum (32). Our analysis revealed the presence of 3 prominent clefts in MglC (Fig. 3*A*). Cleft 1 is the largest among all and is formed at the interface of the dimer with a volume of ∼3946 Å^3^. This cleft 1 includes highly conserved positively charged residues imparting a net positive charge to the region as revealed by the ConSurf analysis and electrostatic potential map, respectively (Fig. 3, *B* and *C*) (33, 34, 35, 36, 37, 38). The clefts 2 and 2′ (the prime is used for identical cleft on another protomer in the dimer) include the ‘FDI’ region shown to be involved in binding MglB and have a volume of ∼580 Å^3^. The clefts 3 and 3′ have ∼600 Å^3^ volume and are lined by variable residues. So, these clefts may be the probable sites for mediating interactions with the binding partners. ConSurf (33, 34, 35, 36, 37) and MSA analysis revealed high conservation in the turn region (F26 to I29) connecting β1-β2 strands, and these residues (F26, D27, and I29) have been previously shown to be involved in binding MglB (Fig. 3, *B* and *C*). The other highly conserved regions are highlighted in Figure 3*B*.

### MglC has distinct structural features among RLC7 family proteins

**Figure 4:**
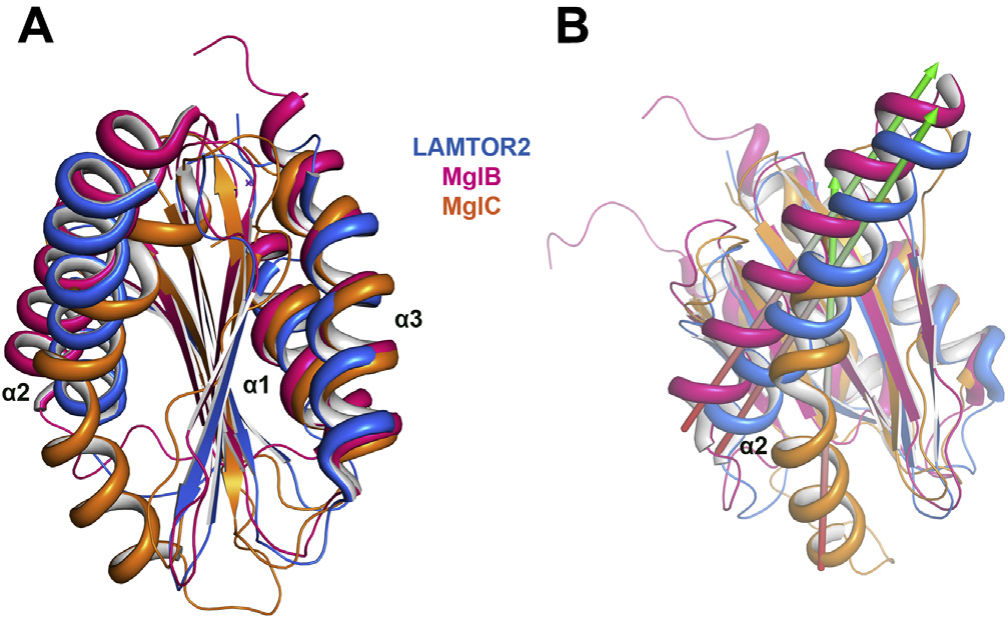
**Comparative structural analysis of MglC with other RLC7 family proteins.***A*, structural superposition of MglC in *orange* with other proteins of RLC7 family LAMTOR2 (40) (PDB ID: 5Y3A, *blue*), and MglB from *Myxococcus xanthus* (19), (PDB ID: 6H5B, *pink*). *B*, structural comparison of MglC showing difference in relative orientation of α2 compared with other RLC7 family proteins. MglB, Mutual gliding-motility protein B; MglC, Mutual gliding motility protein C; RLC7, Regulatory Light Chain 7.

We searched for proteins sharing structural similarity with MglC using PDBeFold (39). The top hit included late endosomal/lysosomal adaptor and MAPK and MTOR activator 2 (LAMTOR2) (PDB ID: 5Y3A) (40) protein having 1.88 Å r.m.s.d. over 94 residues sharing 10% sequence identity. Interestingly, MglB (PDB ID: 6HJM) (19) shares ∼9% sequence identity and is among the top hits having 1.91 Å r.m.s.d. over 93 residues. The top hits obtained from the PDBeFold (39), and structural comparisons are provided in Tables S1 and S2. MglB α2 is functionally important and has been shown to interact with switch 1 and switch 2 regions of MglA (19, 20). Comparative structural analysis revealed that α2 in MglC is drastically shifted as compared with MglB and other RLC7 fold proteins (Fig. 4, Fig. S3). For example, α2 helix of MglC is tilted by ∼40° and ∼33° having a maximal displacement of 17.7 Å and 13.8 Å compared with α2 of MglB (6HJM, *M. xanthus*) and LAMTOR2 (5Y3A, *Homo sapiens*) (19, 40), respectively (Fig. 4). The relative shift in α2 of MglC compared with α2 of other RLC7 family proteins is given in Table S1. In addition, there is a presence of 3_10_ helix connecting α2 and β2 and highly conserved G67 that is present only in MglC. Upon comparing MglC dimer with the dimers of other RLC7 family members, we also observed that α1 and α3 helices are shifted slightly inward in MglC. For example, compared with *M. xanthus* MglB α1, MglC α1 is shifted by an angle of ∼7.2° and displacement of 3.64 Å, while compared with *H. sapiens* LAMTOR2 α1, it is shifted by an angle of ∼14.6° and displacement of 4.86 Å. The α3 helix of MglC is also shifted by an angle of ∼15.9° and ∼10.7° and displacement of 2.73 Å and 3.44 Å compared with *M. xanthus* MglB and *H. sapiens* LAMTOR2 (Fig. S3). This comparative structural analysis reveals similarities and distinct structural features in MglC compared with other members of RLC7 family proteins. The comparative r.m.s.d. analysis of MglC homodimer with dimers of RLC7 family proteins is shown in Fig. S3 and Table S2.

### MglC interacts with MglB with submicromolar range dissociation constant

**Figure 5:**
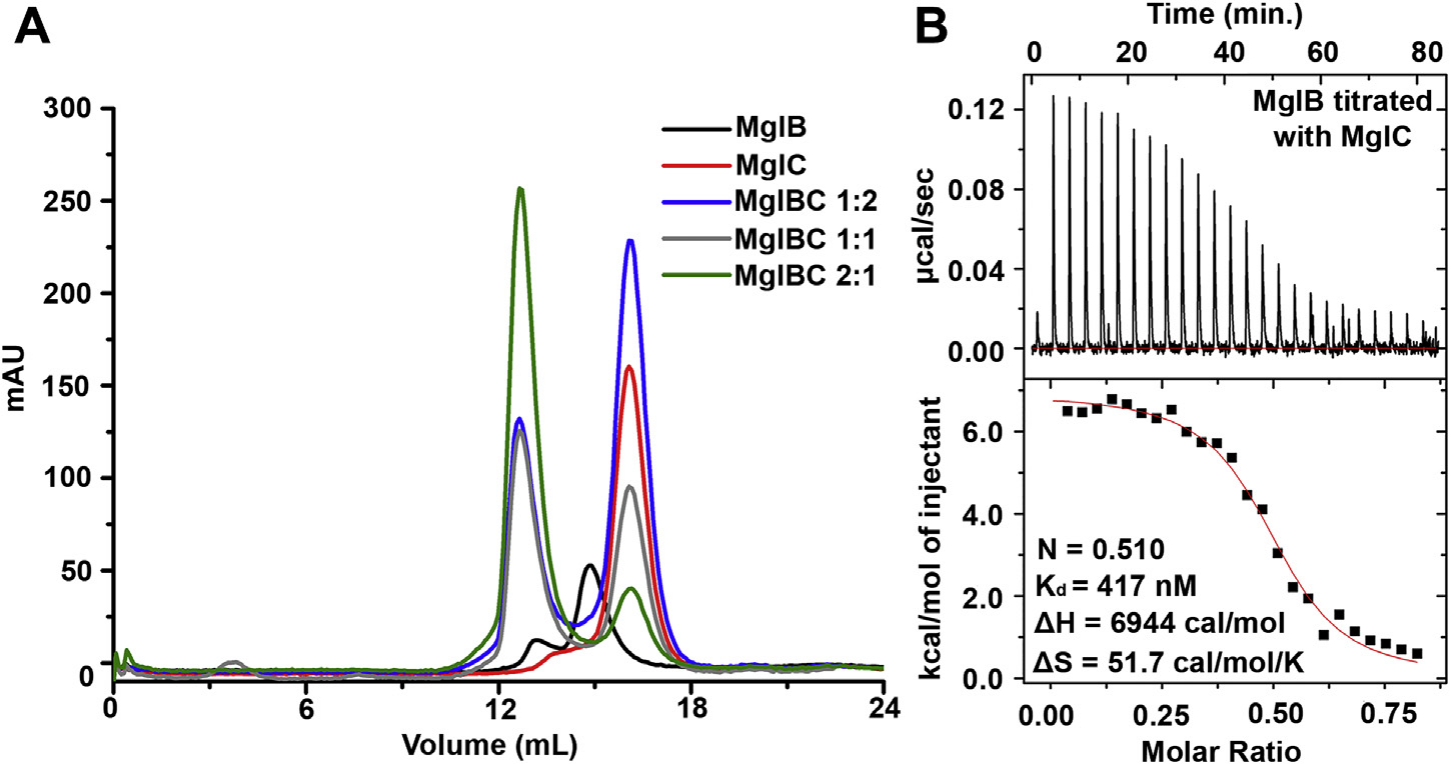
**Interaction of MglC with MglB.***A*, analytical size-exclusion chromatography profile showing the interaction of MglB with MglC. *B*, ITC profile of MglB–MglC interactions. ITC experiments were performed in triplicates. We observed binding stoichiometry of 0.51 ± 0.03, K_d_ of 417 ± 179 nM, ΔH of 6944 cal/mol, and ΔS of 51.7 cal/mol/K. ITC, isothermal titration calorimetry; MglB, Mutual gliding-motility protein B; MglC, Mutual gliding motility protein C.

Using bacterial two-hybrid assay, McLoon *et al.* (29) have proposed that MglC might interact with MglB *via* the ‘FDI’ interface. To check physical interactions between MglB and MglC, we mixed both the purified proteins in varying ratios and performed analytical SEC experiments. MglB and MglC were eluted predominantly at ∼15 ml and ∼17 ml corresponding to the observed molecular weights of ∼42 kDa and ∼35 kDa (MglB, Mol. Wt. 20.01 kDa; MglC, Mol. Wt. 15.94 kDa), respectively, suggesting both proteins exist as homodimers in the solution (Fig. 5*A*). When we mixed both the proteins in the equimolar ratio, we obtained two peaks corresponding to the MglBC complex (Mol. Wt. 112.28 kDa) at ∼13 ml and excess MglC alone at ∼17 ml, confirming interactions of MglC with MglB (Fig. 5*A*). When MglB:MglC were mixed in 2:1 ratio, we observed only a small MglC peak, whereas the majority of the sample eluted as an MglBC complex. However, when MglB:MglC were mixed in 1:2 ratio, there was no increase in the intensity of the MglBC complex peak, whereas the excess MglC peak was observed at 17 ml (Fig. 5*A*). This suggested that MglC binds MglB in 2:4 stoichiometry (as both proteins exist in a homodimeric state in the solution) to form the MglBC complex. The MglBC complex was stable during the SEC run, suggesting MglC binds MglB with high affinity. Therefore, we performed ITC experiments to determine binding affinity and stoichiometry of MglB–MglC interactions. We used one site model for data fitting. ITC data revealed K_d_ of 417 ± 179 nM and stoichiometry (N) of 0.51 ± 0.03, hence confirming that two homodimeric MglB molecules bind one homodimeric MglC (Fig. 5*B*). As the enthalpy (ΔH) is positive 6944 cal/mol, that is, endothermic process, along with positive entropy (ΔS) 51.7 cal/mol/K, it is suggested that the reaction is entropy driven.

### The C-terminal region of MglB is not involved in binding MglC

The crystal structure of the MglAB complex revealed involvement of the C-terminal region (residue 147–157 of the MglB protomer) of MglB in binding MglA. This was further confirmed by deletion studies (20). In the MglAB complex, the C-terminal region of only one protomer is involved in binding MglA (20). To investigate the role of this C-terminal region of MglB in binding MglC, we created a deletion variant of MglB (MglB^ΔCT^). We performed SEC based protein–protein interaction studies using this deletion variant. Our data suggest that the MglB deletion construct retains the ability to bind MglC (Fig. S4). These data suggest the MglB may adopt a distinct mode of binding MglC to form the MglBC complex compared with the MglAB complex.

### MglC homodimer is sandwiched between two MglB homodimers

**Figure 6:**
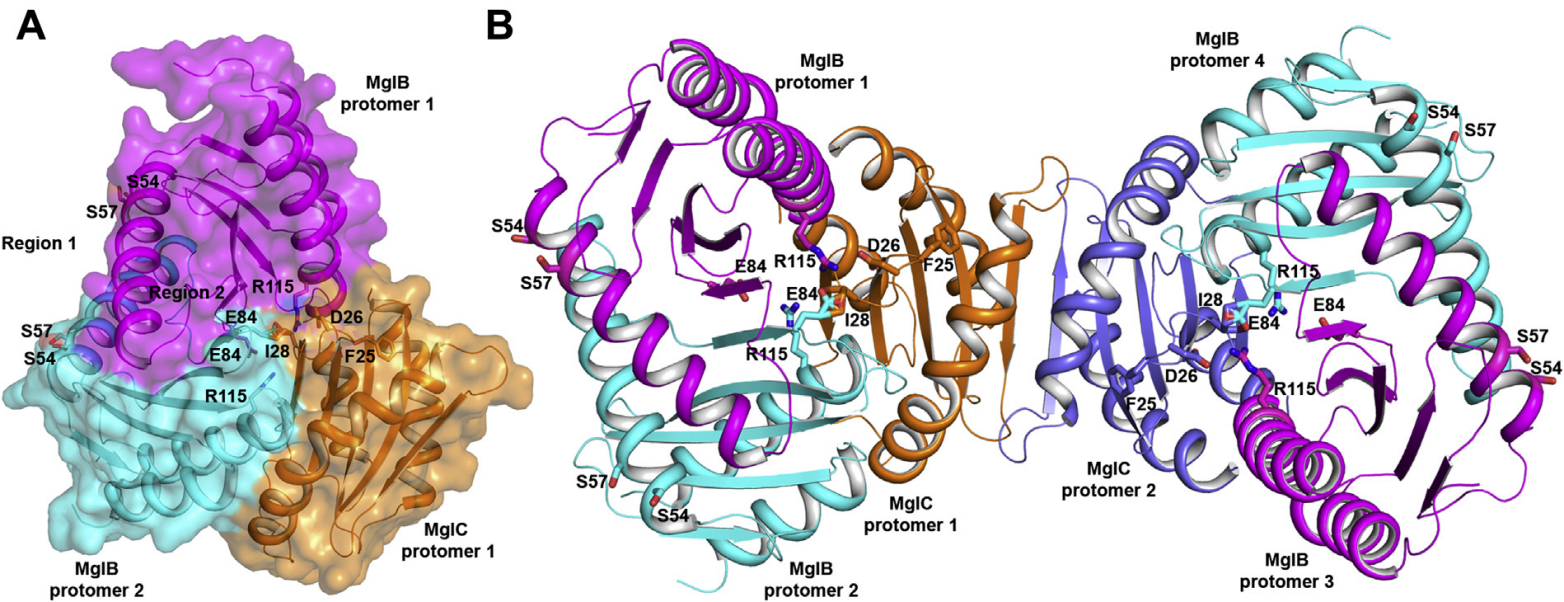
**MglC interaction interface with MglB.***A*, MglC binds MglB with the FDI interface. The model obtained from molecular docking of MglC with MglB shows that the FDI interface (*black dotted circle*) of MglC is involved in binding MglB. *B*, proposed model for the MglBC complex based on experimentally determined binding stoichiometry and docking results. MglB, Mutual gliding-motility protein B; MglC, Mutual gliding motility protein C.

**Figure 7:**
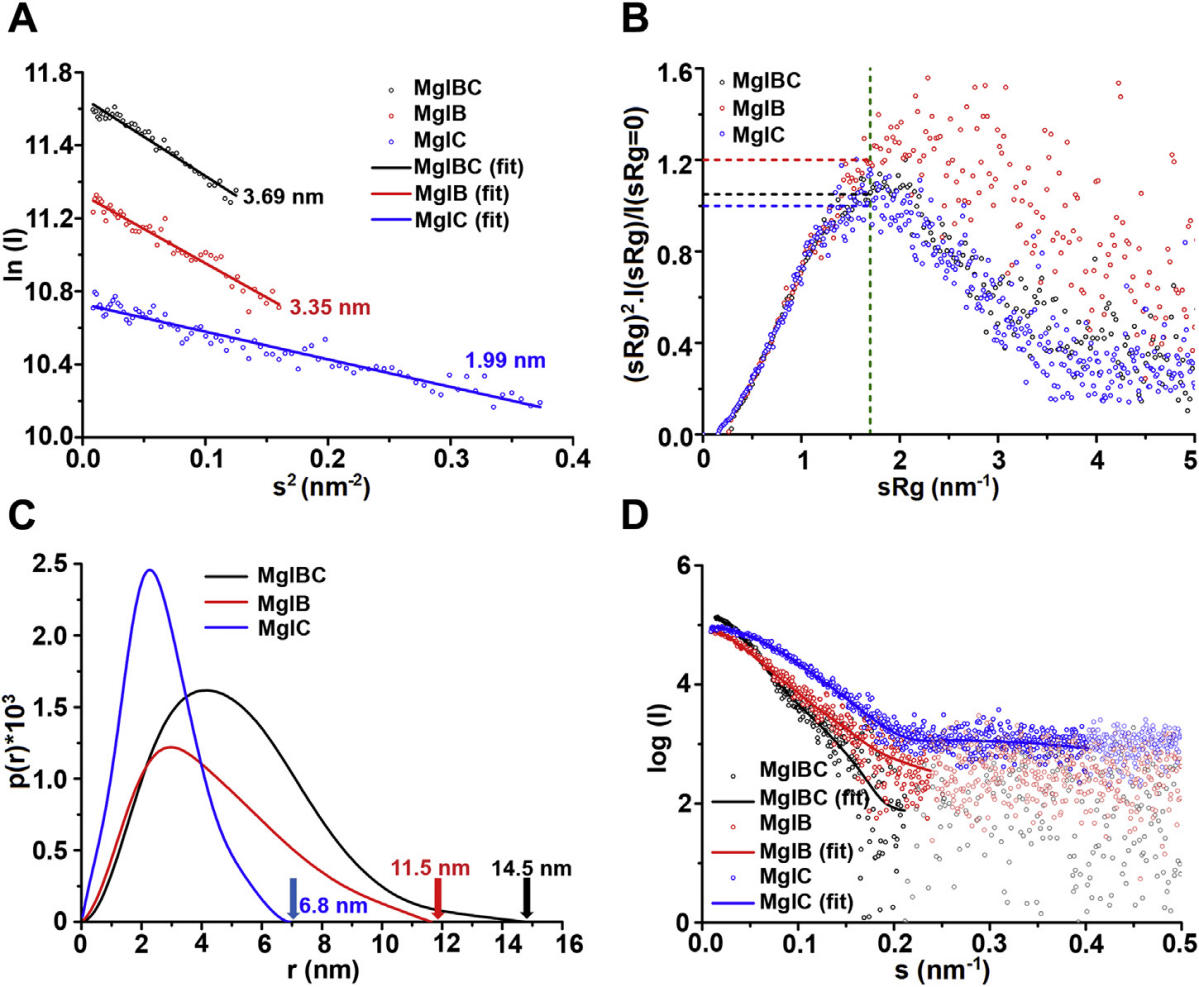
**SAXS analysis of MglC (*blue*), MglB (*red*), and MglBC complex (*black*).***A*, Guinier analysis of MglC (Rg = 1.99 nm, R^2^ = 0.933), MglB (Rg = 3.35 nm, R^2^ = 0.938), and MglBC (Rg = 3.69 nm, R^2^ = 0.959) complex reveals linear fit with no signs of interparticle effects. *B*, dimensionless Kratky plots of MglC and MglBC complex revealed globular nature of these proteins. The Kratky plot revealed that the MglB was folded and had some flexible regions. *C*, normalized pair distribution function P(r) analysis revealed D_max_ of ∼6.8 nm for MglC, 11.5 nm for MglB, and ∼14.5 nm for the MglBC complex. *D*, the dummy atom model for MglC (χ^2^ = 0.81), MglB (χ^2^ = 0.71), and MglBC (χ^2^ = 0.60) complex were prepared using GASBOR (44). The intensity profile of all three proteins is shown as *spheres*, and the *lines* represent the fitting of dummy atom models generated by GASBOR (44). MglB, Mutual gliding-motility protein B; MglC, Mutual gliding motility protein C; SAXS, small-angle X-ray scattering.

The studies presented above demonstrated that MglBC forms a stable complex in the solution. We performed extensive crystallization experiments to determine the structure of the MglBC complex. We successfully crystallized the complex; however, we did not succeed in improving the diffraction quality of the crystals. So, we next performed protein–protein docking using the ClusPro 2.0 server. Several models were generated by this server. The top ten models were shortlisted for manual analysis. McLoon *et al.* (29) have previously shown that MglB binds the ‘FDI’ surface of MglC. Interestingly, in all the docked structures from ClusPro 2.0 server (41, 42, 43), we observed that MglB was indeed docked at the FDI interface on MglC (Fig. 6). The ‘FDI’ interface is a part of negatively charged residues containing cleft 2 as described in the previous section. None of the models obtained from the docking servers showed involvement of the C-terminal region of MglB in binding MglC, which correlates well with our experimental findings. We also performed ConSurf and Electrostatic surface potential analysis of MglB (PDB ID: 6HJM). We observed that the MglC binding face of MglB is highly conserved and positively charged (Fig. S5). Therefore, probably ionic interactions may be involved in stabilizing the MglBC complex. Based on the experimentally determined binding stoichiometry and docking results, we generated a model of the MglBC complex where the MglC homodimer is sandwiched between the two MglB homodimers (Fig. 7). In this proposed model, the MglB homodimer interacts with one chain of MglC, and the complex is related by two-fold symmetry.

### SAXS-based low-resolution in solution structure of the MglBC complex

**Figure 8:**
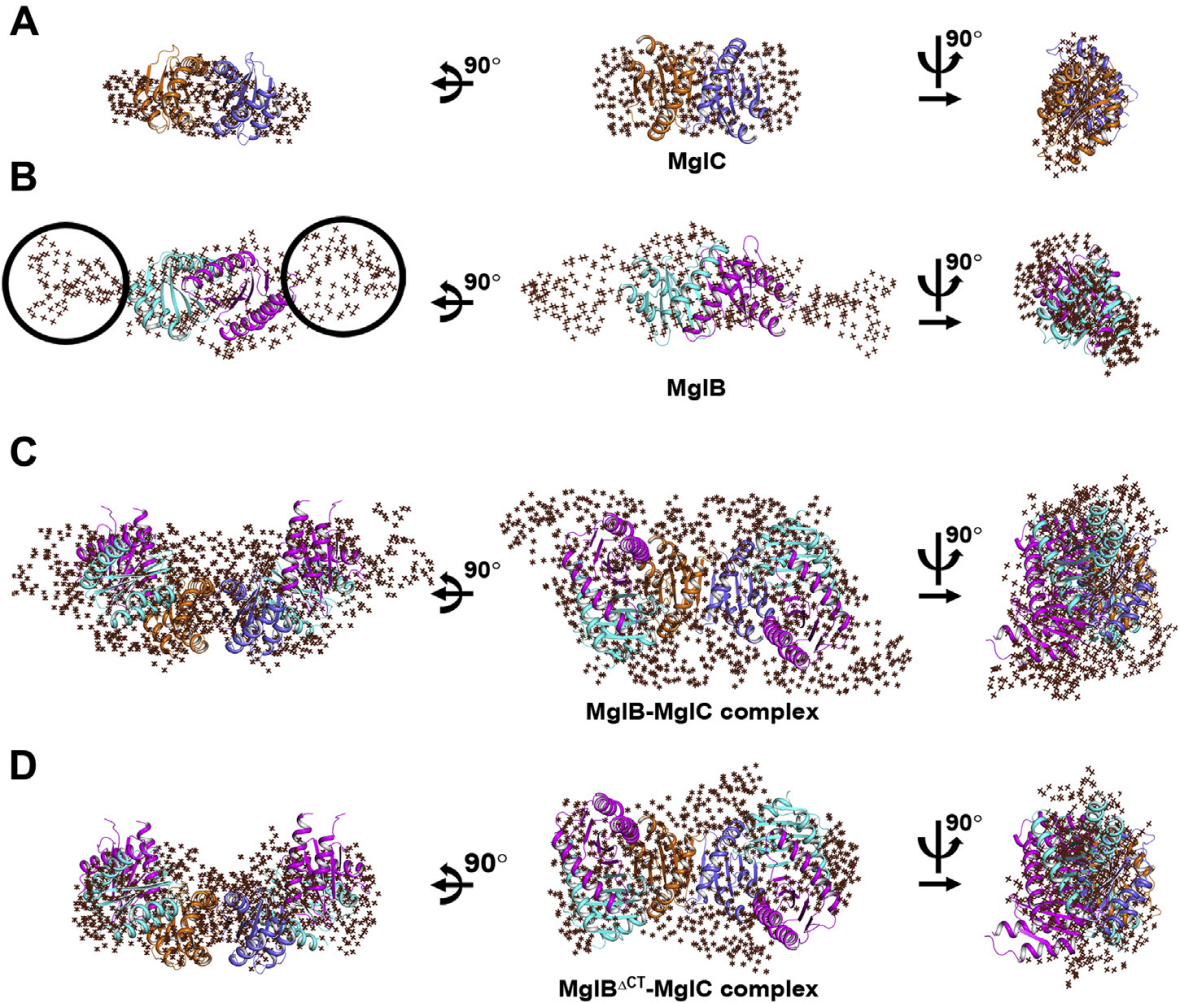
**Low-resolution solution structure of MglC, MglB, and MglBC complex fitted in the SASX envelope using high-resolution crystals structures and modeled regions not resolved in crystal structures.***A*, the MglC homodimer superposes well in the dummy model. *B*, the MglB homodimer core fits well. The extra regions in the SAXS envelope are probably due to the flexible N- and C-terminal regions in MglB. *C*, low resolution structure of the MglBC complex obtained by molecular docking of MglC with MglB reveals the presence of MglC dimer sandwiched between two molecules of the MglB dimer. The high-resolution models were fitted in the low-resolution models using SUPCOMB (47). The unaccounted regions in the dummy atom model suggest the presence of flexible regions not resolved well in the crystal structures. MglB, Mutual gliding-motility protein B; MglC, Mutual gliding motility protein C; SAXS, small-angle X-ray scattering.

To further confirm oligomeric status, binding stoichiometry, and the predicted MglBC complex model, we performed SAXS experiments. We collected SAXS data on three different concentrations of unliganded MglB, and MglC, and their complex, MglBC as described in the Experimental procedures section. All data collected for the samples were free from interparticle effects or aggregation. Guinier analysis considering globular scattering profile estimated the radius of gyration (R_g_) values of 3.35 nm, 1.99 nm, and 3.69 nm for MglB, MglC, and MglBC, respectively (Fig. 7*A*). Dimensional Kratky analysis of all the proteins suggested that all the proteins were folded, and the first maxima was at 1.7 for unliganded MglC and MglBC, and for MglB, the maxima was >1.7, implying partly disordered portion in its shape (Fig. 7*B*). The maximum linear dimension (D_max_) obtained for MglB, MglC, and MglBC were 11.5 nm, 6.8 nm, and 14.5 nm, respectively (Fig. 7*C*). The molecular weight was calculated by dividing the Porod volume (∼89,089 Å^3^, ∼47,239 Å^3^, and ∼207,733 Å^3^ for MglB, MglC, and MglBC, respectively) by 1.7. This calculated molecular weight to be ∼52 kDa, ∼28 kDa, and ∼122 kDa of MglB, MglC, and MglBC, respectively, which were found to be in close agreement with the theoretical molecular weights for homodimeric MglB (∼40 kDa), homodimeric MglC (∼32 kDa), and 4:2 stoichiometric MglBC complex (∼112 kDa). All structural parameters for MglC, MglB, and MglBC complexes are given in Table S3. The dummy-atom models were then built using GASBOR (44). CRYSOL analysis of the top ten ClusPro models generated for the MglBC complex with the SAXS data of the MglBC complex is shown in Fig. S8*A* (45, 46). The χ^2^ of 2.109 ± 0.16 was obtained when the MglBC complex ClusPro-generated models were fitted into the experimental SAXS data. The fitting of one of the GASBOR-generated models with an intensity profile is shown in Figure 7*D* and Fig. S8*C*. These models were then aligned to the crystal structures of MglC and MglB, and molecular docking–generated models *via* SUPCOMB, which aligns inertial axes of the models (47) (Fig. 8, Fig. S7). Residue resolution structure of MglC aligned well with the shape profile solved using the SAXS data–based constraints (Fig. 8*A*, Fig. S7*A*). However, models generated for the MglB and MglBC complex revealed extra regions that were not resolved in the crystal structure of MglB, probably because of inherent flexibility or accessibility to different local conformations relative to the structure domain (Fig. 8*B*, Fig. S7*B*, Fig. 8*C* and Fig. S7*C*). We observed the number of Shannon channel for GASBOR-generated models to be 8.891, 8.849, and 13.19 for MglC, MglB, and MglBC, respectively. Normalized spatial discrepancy (NSD) values for various GASBOR-generated models for the MglC, MglB, and MglBC complexes are 0.855 ± 0.019, 1.586 ± 0.051, and 1.646 ± 0.137, respectively, showing low spatial discrepancy between GASBOR-generated models. To further investigate the role of the C-terminal region in protein–protein interactions in the MglBC complex, we performed SAXS experiments using MglB^ΔCT^ as well. The SAXS data suggest that MglB^ΔCT^C forms a complex with R_g_ of 3.16 nm and D_max_ of 10.4 nm (Fig. 8*D*, Fig. S7*D*, and Fig. S6), further supporting data obtained using SEC. The number of Shannon channel calculated for the GASBOR-generated model of MglB^ΔCT^C was 8.358. NSD for the GASBOR-generated model of the MglB^ΔCT^C complex is 1.319 ± 0.047. All structural parameters for the MglB^ΔCT^C complex are given in Table S3. CRYSOL analysis of the ten ClusPro models generated for the MglBC complex with the SAXS data of the MglB^ΔCT^C complex is shown in Fig. S8*B* (45, 46). The χ^2^ of 2.107 ± 0.027 was obtained after CRYSOL analysis of the MglB^ΔCT^C complex with its corresponding SAXS data. So, the SAXS experiments confirmed the oligomeric states of the MglB, MglC, and MglBC protein–protein complex. Also, these experiments further strengthen a molecular docking–based model proposed for the MglBC complex, where probably the MglC homodimer is sandwiched between the two MglB homodimers (Fig. 6).

### Site-directed mutagenesis confirms the MglB–MglC binding interface

To validate the MglB–MglC binding interface obtained by molecular docking studies, we created MglB and MglC mutants using site-directed mutagenesis. We created MglB mutants at two distinct regions, that is, region 1 and region 2. We created the MglB^E84A,R115A^ variant targeting conserved residues at the interface involved in binding MglC predicted based on molecular docking studies. The MglB^S54A,S57A^ variant encompasses conserved residues that are shown previously to interact with MglA. CD spectroscopy studies suggest that the MglB mutants are well folded, and their comparative analysis shows no changes in the secondary structural contents of MglB mutants (Fig. S9, Table S4). We then mixed MglB^E84A,R115A^ and MglB^S54A,S57A^ with MglC, respectively, in 2:1 ratio and observed that MglB^E84A,R115A^ mutation resulted in weakening of the MglB/C interface as observed by the reduction in the MglBC complex population in SEC (Fig. S9). Although the MglB/MglC complex formation is not affected by MglB^S54A,S57A^ mutation. This observation confirmed that the MglB^E84A,R115A^ interface is involved in complex formation, which is distinct from the binding interface shown to be involved in binding MglA.

### MglC is not recruited at poles by itself

Galicia *et al.* (19) have previously shown that MglB contains a stretch of positively charged surface but it cannot bind to liposomes. Molecular docking and site-directed mutagenesis–based experiments in this study suggest that this positively charged region in MglB forms the MglB–MglC binding interface. Like MglB, electrostatic surface analysis of MglC showed the presence of conserved positively charged surface at cleft 1. We therefore wanted to check whether MglC interacts with negatively charged lipids, that is, cardiolipins that are present mainly at bacterial poles. However, like MglB, we also did not observe interaction of MglB, or MglC or MglBC complex with liposomes (Fig. S10). This suggests that MglC is recruited to poles by the help of an interacting partner such as RomR as proposed by McLoon *et al.*

## Discussion

Cellular polarity reversal is an important phenomenon required for various essential functions for existence such as motility, development, biofilm formation, and many more (8, 48). MglC is a newly discovered and the least studied member involved in polarity reversal. There is only one publication describing the discovery and role of MglC in polarity reversal. In this study, we determined the crystal structure of a recently identified member of the polarity reversal complex, MglC (Fig. 2). Crystal structure MglC revealed structural similarity with MglB, hence confirming both are members of the RLC7 protein family (Fig. 4, Fig. S3). Despite sharing similar fold architecture, there are distinct structural features in MglC, including the deletion of N- and C-terminal regions and differences in the overall arrangement of the secondary structural elements, which might dictate preferences for specific binding partners. We also show that MglC binds MglB with 2:4 stoichiometry, respectively, to form a stable complex with submicromolar range dissociation constant (Figs. 5 and 6).

Koonin and Aravind (49) have previously shown the presence of invariant glycine that is preceded by a negatively charged residue in the turn between β1 and β2 of RLC7 family proteins. They further proposed that this turn is critically involved in the functioning of these proteins. In MglC crystal structure, we also observed that the “FDGI” region involved in binding MglB is present between the turn connecting β1 and β2 (Fig. 6). Furthermore, crystal structures of various heteropentameric ragulator complexes (*e.g.*, LAMTOR 1–5 in *H. sapiens*) have been determined (40, 50). Unlike MglB and MglC, human ragulator complexes LAMTOR4-5 form heterodimers and they interact with LAMTOR2-3 heterodimer *via* a turn between β1 and β2 of LAMTOR5 only (40, 50), although this structurally equivalent region of LAMTOR4 is free. Therefore, LAMTOR2-3 binds LAMTOR4-5 in 2:2 stoichiometry. Probably, other LAMTOR2-3 cannot bind because of steric clashes that might occur if LAMTOR4-5 and LAMTOR2-3 binds in 2:4 stoichiometry. This loop region is shifted in MglC probably making the sterically favorable environment for 2:4 binding stoichiometry.

Cleft analysis of MglC suggested the presence of three distinct clefts, that is, 1, 2 (and 2′), and 3 (and 3′) in each protomer (Fig. 3). Molecular protein–protein docking results obtained from ClusPro 2.0 (41, 42, 43) suggest the involvement of cleft 2 and 2’ in binding MglB. We used SAXS-based analysis to further obtain low-resolution insights to propose a model for MglBC complex. MglC cleft 2 contains highly conserved ‘FDI’ sequence motif that has been shown to be involved in binding MglB (29).

MglC also contains cleft 1 with a convex shape that contains conserved and positively charged surface. We therefore performed liposome cosedimentation assay to check the interaction of MglC with liposomes. However, we did not observe the interaction of MglC with liposomes (Fig. S9). McLoon *et al.* have previously suggested that MglC is probably recruited to poles in an asymmetric manner by RomR. Because our molecular docking and mutagenesis experiments suggest that this positively charged region in MglB is probably involved in binding MglC, these interfaces may not probably be involved in binding lipid membrane. So, the polar localization of MglB and MglC may be dependent on the interacting protein partner(s) (such as RomR) as proposed earlier.

**Figure 9:**
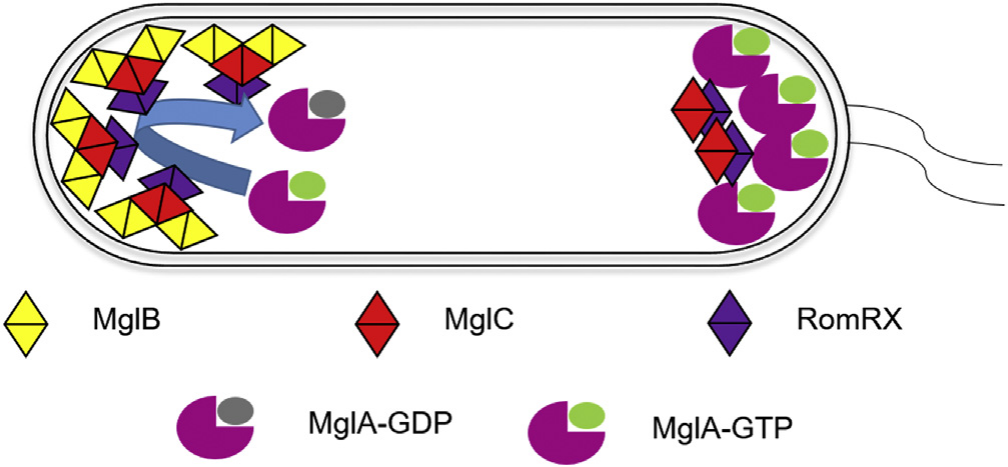
**Model for regulation of polarity reversal in *Myxococcus xanthus*.** According to our proposed model, RomR recruits MglC to poles in an asymmetric manner, and at the lagging pole, MglC binds MglB in 2:4 stoichiometry, whereas MglA is present at the leading pole. The polarity reversal starts as the MglA and MglB relocate followed by RomRX and MglC. MglA, Mutual gliding-motility protein A; MglB, Mutual gliding-motility protein B; MglC, Mutual gliding motility protein C; RomRX, Required for motility response regulator complex.

Based on previous studies on polarity reversal pathway of *M. xanthus* and data presented in this study, we propose a model for cellular polarity reversal (Fig. 9). We propose that the recruitment of MglB, MglC, or MglBC complex to the poles is mediated by binding to membrane-localized interacting partner(s) such as RomRX. RomRX is recruited to the poles in a bipolar asymmetric manner and recruits MglC in a similar manner having higher concentrations at the lagging pole (29). Because MglB is present only at the lagging pole, MglC probably interacts to form the MglBC complex only at the lagging pole. However, currently due to lack of data, the mechanism of localization of MglA and MglB at the leading and the lagging poles, respectively, is not clearly understood.

During polarity reversal, MglA and MglB reportedly localize together at the lagging pole for about 30 s (17). Because the formation of the MglAB and MglBC complexes has now been well established, it will be interesting to see if these proteins interact to form the MglABC ternary complex and if this complex has some role in polarity reversal. After MglA has traveled to the opposite pole, MglB is then relocated to the other pole while MglC and RomRX together still remain at the lagging pole (29). This relocation of MglB requires modulation of the MglBC complex, which probably requires Frz signaling. Extensive research is required to understand the dynamics of protein–protein interactions that play critical role in polarity reversal.

MglB reportedly binds to several proteins involved in polarity reversal including MglA, RomRX, SofG, and MglC (19, 20, 22, 29, 51). The MglAB complex has been structurally characterized (19, 20). Based on pull-down experiments of 6x-His–tagged MglB with *M. xanthus* cell lysate, it has been shown that MglB and RomR could potentially interact (16, 52). MglC also reportedly interacts with RomR, but probably at the binding site different from MglB (29). All these studies and the data presented here suggest that both MglB and MglC have multiple binding partners and partner switching may be required for polarity reversal. To our knowledge, the binding affinities for these reported binding partners with MglB or MglC have not yet been determined. Hence, we could not compare affinities with other known complexes involved in polarity reversal. In our binding studies, we observed moderate submicromolar dissociation constant for the MglBC complex. We speculate that this moderate affinity may facilitate partner switching at cellular concentrations. MglA (small GTPase), MglB (GTPase-activating protein), and RomR (guanine exchange factor) are known to be modulated by Frz-mediated phosphorylation (16, 17, 22). It could be possible that Frz signaling also plays significant role in modulating the binding partners of MglC or could modulate the binding affinity of MglC for MglB and RomR. This needs a thorough investigation in the future studies.

To summarize, we report the first structural description of MglC involved in polarity reversal in *M. xanthus*. We have established and characterized the MglBC complex in detail. Our data suggest that MglC exists in a homodimeric oligomeric state in the solution and interacts with two MglB homodimers with a submicromolar range dissociation constant. In future, it will be interesting to know how cellular reversals are regulated by protein–protein interactions involving multiple binding partners. Detailed structural and functional studies will be required to understand the role of protein–protein interactions in mediating polarity reversal.

## Experimental procedures

### MSA

For MSA-based analysis, the protein sequence of MglC (ABF90799.1) was submitted at NCBI BLAST (53) to retrieve sequences sharing sequence similarity. From the BLAST results, ten homologs sharing more than 30% sequence identity were selected for MSA. For comparison with MglB, five MglC and MglB homologs with more than 30% sequence identity were selected for MSA. The MSA was then generated using Constraint-Based multiple Alignment Tool (COBALT) (54). Alignment files and crystal structures of MglC and MglB (PDB ID: 6HJM) were then used in Easy Sequencing in Postscript (ESPript) 3.0 server (55) for generating structure-based sequence alignments.

### Cloning, expression, and purification of MglC and MglB

The *mglC, mglB* and *mglB*^*ΔCT*^ genes were amplified from genomic DNA of *M. xanthus* (DSMZ, catalog number 16526) using primers mglC-F (5′-GCTAGTCGCTAGCTCCTTCCGCACGCACCTCGAG-3′), mglC-R (5′-GCTAAAGCTTCTAGAGCTCGGCGCGCACCT-3′), mglB-F (5′-GCTGAAGCTAGCATGGGCACGCAACTGG-3′), mglB-R (5′-CGTAAAGCTTTTACTCGCTGAAGAGGTTGTCG-3′), mglB^ΔCT^-R (5′-CGTAAAGCTTTTACACCAGGCTCTCGAAGATCTTCGTGAGCTC-3′) synthesized by Sigma-Aldrich. The *mglC*, *mglB*, and *mglB*^*ΔCT*^ PCR products were cloned in pET-Duet-A-TEV, (engineered pET-Duet-1, Novagen, vector with a tobacco etch virus (TEV) cleavage site) with TEV-cleavable 6x-His tag at N-terminal of the gene cloned between NheI (New England Biolabs Inc) and HindIII (New England Biolabs Inc) to yield pET-Duet-A-TEV-mglC, pET-Duet-A-TEV-mglB, and pET-Duet-A-TEV-mglB^ΔCT^ clones. Ligated products were transformed in *Escherichia coli* Top10 cells (Novagen) and were confirmed by DNA sequencing. Plasmids carrying the desired gene(s) were transformed in *E. coli* Rosetta (DE3) cells (Novagen) and plated on LB agar plate having 100 μg ml^−1^ ampicillin (Sisco Research Laboratory Pvt. Ltd) and 35 μg ml^−1^ chloramphenicol (Sisco Research Laboratory Pvt. Ltd) and were incubated overnight at 37 °C. The colonies obtained on the plates were used for protein purification. A single colony was inoculated in 10-ml LB media and incubated overnight with the constant shaking of 200 r.p.m. at 37 °C for primary culture. 1% of primary culture was inoculated in 750 ml of LB media and induced with 0.3-mM IPTG (Gold Biotechnology) when O.D._600nm_ reached ∼0.6 and further incubated at 16 °C for 14 to 16 h with constant shaking at 200 r.p.m. Cells were harvested by centrifugation at 10,000*g* for 10 min. The pellet was then resuspended in 50-ml lysis buffer (20-mM HEPES, pH 8.0, 150-mM NaCl). Protease inhibitor cocktail tablets (Roche) were added to the lysis buffer before sonication. The supernatant was collected after centrifugation at 18,000*g* for 30 min and passed through pre-equilibrated HIS-Select Ni-nitrilotriacetic acid resin (Sigma-Aldrich Co) at 4 °C for binding of 6x-His–tagged protein. The protein was then eluted with the lysis buffer containing imidazole (Sigma-Aldrich Co) at different concentrations (20 mM, 200 mM, and 500 mM) and concentrated using centrifugal ultrafiltration devices (3-kDa cut-off) (Merck India Pvt. Ltd). This was followed by SEC using Superdex 200 Increase 10/300 GL column (GE Lifesciences) with a flow rate of 0.5 ml per min. The desired fractions were pooled and concentrated using centrifugal ultrafiltration devices (Merck India Pvt. Ltd). The purity and quality of purified protein samples were checked using SDS-PAGE, and the concentration was measured using bicinchoninic acid protein assay (Thermo Scientific).

The purification tag was removed by incubating protein samples with TEV protease in a protease:protein ratio of 1:30 and incubated at 4 °C for 16 h. The protein digestion was checked on SDS-PAGE, and cleaved protein was further purified by SEC using a Superdex 200 Increase 10/300 GL (GE Lifesciences) column and concentrated using centrifugal ultrafiltration devices.

### Site-directed mutagenesis, expression, and purification of MglB

MglB double mutants were created after analyzing the surface interacting with MglC and MglA. Site-directed mutagenesis was performed to create two double mutants, MglB^E84A,R115A^ and MglB^S54A,S57A^. Single primers were designed to construct the mutants: MglB^E84A^ primer, CGAGTTCCCCAACCAGTTCCACGCCGGGGCCAAGGACTCGCTG, was used to incorporate E84A mutation, and the obtained construct was further used to incorporate R115A mutation using MglB^R115A^ primer 5′-CCAGCCTCGGCCTCGTCGCCCTTCGCATCAAGAAGGCCAGCG-3′. MglB^S54A,S57A^ mutant was generated using primer 5′ -GACGCAGAACATCGACACCACGGCCCTGGCCGCCCTGACGGCCGGTAACGTGGCCG-3′. PCR amplification using the above designed primers was performed using pET-Duet-A-TEV-mglB as a template. The obtained PCR product was further digested using DpnI enzyme (ThermoFisher Scientific) and transformed into *E. coli* Top10 cells (Novagen). The colonies obtained were used to inoculate a 10-ml culture that was grown overnight at 37 °C. Plasmid isolation was performed using the plasmid isolation kit (Thermo Fisher Scientific). The incorporation of mutation was confirmed by DNA sequencing. The mutants were then transformed in *E. coli* Rosetta (DE3) cells (Novagen), and proteins were purified as described above for MglB WT.

### CD spectroscopy of MglB and its mutants

To check the effect of mutation on MglB, we performed CD spectroscopy using Jasco J-815 instrument with data pitch of 1 nm, scanning speed of 50 nm/min, and 5 accumulations per run. Experiments were performed using 17.8 μM of protein sample diluted in type I water. Spectrum for protein samples was measured from 190 to 250 nm wavelength at 25 °C after subtracting blank. The secondary structural content of all the samples was then analyzed using BeSTSel server (56, 57).

### Preparation of selenomethionine-containing protein for phasing experiments

For incorporation of selenomethionine (Sigma-Aldrich Co) into the protein, minimal media was used to grow *E. coli* Rosetta (DE3) cells (Novagen) harboring expression plasmid coding for MglC. The medium was composed of M9 salts (Na_2_HPO_4_.7H_2_O, 33.97 g/l, KH_2_PO_4_, 15 g/l, NaCl, 2.5 g/l, NH_4_Cl, 5 g/l, (Sigma-Aldrich Co), 1 M MgSO_4_ (Sigma-Aldrich Co), 1 M CaCl_2_ (Sigma-Aldrich Co), 20% glucose (Sigma-Aldrich Co), trace elements, and amino acids (threonine 100 mg/l, phenylalanine 100 mg/l, lysine 100 mg/l, isoleucine 50 mg/l, valine 50 mg/l, selenomethionine 60 mg/l) (Sigma-Aldrich Co)). All components of minimal media were prepared in Milli Q water and sterilized separately. Glucose, trace elements, and amino acids were filter-sterilized, and all other components were autoclaved. To prepare the media (1 l), 200 ml of M9 salts, 2 ml 1 M MgSO_4_, 20-ml glucose, 100-ml 1 M CaCl_2_, and 1x trace elements were mixed together and the volume was made up to 1 l using autoclaved water. The primary culture was grown in the similar fashion as mentioned above. The cells from primary culture were harvested in the log phase by centrifugation at 4000 r.p.m. for 10 min. The pellet was resuspended in the minimal media and used as inoculum for the secondary culture. The culture was incubated at 37 °C with shaking at 200 r.p.m. Amino acids were added to the culture at an O.D._600nm_ of ∼ 0.6, and the culture was induced by adding 0.3-mM IPTG and incubated at 16 °C for 18 to 20 h. Cells were harvested by centrifugation at 9000*g* for 10 min, and protein was purified as described in the previous section.

### Crystallization and structure solution

Crystallization trials of MglC were set up with TEV cleaved and uncleaved protein samples (9 mg/ml, 12 mg/ml, and 15 mg/ml). Crystallization trials were performed in 3-well high-throughput crystallization plates (Swissci, Hampton Research, Aliso Viejo, CA) using commercial crystallization screens (Hampton Research and Molecular Dimensions, UK). The plates were incubated at 18 °C in RockImager RI1000 (Formulatrix, Inc). Initial hits were observed after 12 h of setting up the crystallization trials in various conditions for cleaved and after 2 days for uncleaved protein samples. The crystals of uncleaved proteins diffracted anisotropically. In contrast, the crystals for TEV cleaved proteins diffracted isotropically. We also produced selenomethionine derivatized MglC crystals. The native and Se-SAD data were collected at Elettra synchrotron radiation source, Trieste, Italy. The data were processed using iMosflm (58) and scaled using AIMLESS in CCP4 software suite (59). The crystal structure was determined by Se-SAD method using Phenix.AutoSol module in the Phenix software suite (60). The structure was further improved using several iterative cycles of manual model building in Coot and refinement using Refmac (61, 62, 63, 64, 65). The final model had R_Work_/R_Free_ of 15.9/21.5. The model obtained from the Se-SAD phasing method was used to solve the crystal structure of native MglC by molecular replacement using Phaser (66). The model obtained from Phaser was further refined by several iterative cycles of model building by Coot and refinement using Refmac (61, 62, 63, 64, 65, 66). The final model for native crystal had R_Work_/R_Free_ of 19.7/23.1. The detailed data collection statistics, model refinement parameters, and validation statistics are provided in Table 1.

**Table 1:** Data collection and refinement statistics

Data collection/refinement statistics of MglC	MglC-SeMet (PDB ID: 7CT3)	MglC-native (PDB ID: 7CY1)
Data collection and processing		
Beamline	Elettra 11.2C λ = 0.97800	Elettra 11.2C λ = 0.98000
Resolution range (Å)^a^	27.52–1.85 (1.916–1.85)	27.98–2.192 (2.27–2.192)
Space group	P 6_5_ 2 2	P 6_5_ 2 2
a, b, c (Å)	96.68, 96.68, 58.28	96.91, 96.91, 58.05
α, β, γ (°)	90, 90, 120	90, 90, 120
Total reflections^a^	226,964 (14,252)	154,682 (11,159)
Unique reflections^a^	14,178 (845)	8534 (708)
Multiplicity^a^	16.0 (16.9)	18.1 (15.8)
Completeness (%)^a^	99.9 (99.6)	98.8 (97.2)
Mean I/sigma (I) a	21.3 (2.29)	26.1 (3.9)
Wilson B-factor	29.82	42.6
R-merge^a^	0.086 (1.455)	0.075 (0.959)
CC_half_^a^	1.000 (0.757)	1.000 (0.885)
Refinement		
R_work_/R_free_	0.159/0.215	0.197/0.231
r.m.s.d. (bonds) (Å)	0.013	0.013
r.m.s.d. (angles) (°)	1.67	1.79
Number of nonhydrogen atoms	991	962
Macromolecules	921	921
Ligands	1	1
Water	69	40
Protein residues	120	120
Average B-factor (Å^2^)	40.06	51.36
Macromolecules	39.09	51.19
Ligand	32.27	45.21
Solvent	53.10	55.32
Ramachandran favored (%)	98.31	99.15
Ramachandran allowed (%)	1.69	0.85

MglC, Mutual gliding motility protein C.

a Statistics for the highest resolution shell are shown in parentheses.

### Analytical SEC

MglB, MglC, and MglB/MglC (mixed in different ratios, *i.e.*, 1:1, 2:1, and 1:2) were resolved by analytical SEC using Superdex 200 Increase 10/300 (GE Lifesciences) column with a flow rate of 0.5 ml/min. The absorption was recorded at 280 nm. The data were plotted using Origin 2016 Software suite (OriginLab Corporation). To confirm the MglB/MglC binding interface created mutants using site-directed mutagenesis, MglB^E84A,R115A^ and MglB^S54A,S57A^ were mixed with MglC, respectively, in 2:1 ratio and were resolved by analytical SEC using Superdex 200 Increase 10/300 (GE Lifesciences) column with a flow rate of 0.5 ml/min. The absorption was recorded at 280 nm. The data were plotted using Origin 2016 Software suite (OriginLab Corporation).

### ITC

ITC experiments were performed at 25 °C with 50 μM of MglB in cell titrated with 500 μM of MglC using MicroCal Auto-iTC200 (Malvern MicroCal, LLC). Twenty-five injections of 0.6 μl (0.1-μl first injection) were given with a spacing of 200 s, and the reference power was kept at 10 μCal/s. The stirring speed of 750 r.p.m. was kept with a filter period of 5 s. Three control experiments were also set up with the same parameters, that is, titration of the buffer alone, titration of MglB with the buffer in a syringe, and buffer titration with MglC in a syringe. All the experiments were performed in triplicates. Data were analyzed using Origin provided with the equipment using one set of sites model (OriginLab Corporation).

### Molecular docking of MglC and MglB

ClusPro 2.0 server (41, 42, 43) was used for docking MglC with MglB. Crystal structures of MglB PDB ID: 6HJM (19) and MglC, determined in this study, were used for the molecular protein–protein docking studies. The top ten docked poses obtained from ClusPro 2.0 server were then further analyzed manually.

### SAXS

SAXS experiments were performed using line collimation on SAXSpace Instrument (Anton Paar, Austria), and data were collected at the CMOS Mythen detector (Dectris). SAXS data were collected on samples of MglB (25, 15, and 10 mg/ml), MglC (35, 15, and 20 mg/ml), and their freshly eluted complex sample from SEC, that is, MglBC complex (18, 9 and 13.5 mg/ml) and MglB^ΔCT^C complex (7, 5 and 2 mg/ml), and their matched buffer. For every run, ∼60 μl of the sample or buffer was exposed for 1 h at 20 °C in a thermostated quartz capillary (1 mm). For all data sets, the position of primary beam was corrected using SAXStreat software. Contribution of buffer components was subtracted, data were desmeared using beam profile, and finally, scattering intensity profile I(q) was saved a function of q, where q is the momentum transfer vector with units in 1/nm. Latter processing was performed using SAXSquant software. The I(q) profiles for the proteins and their complexes were then analyzed and processed using ATSAS 3.0.1 and online versions (46). Using low q data, Guinier analysis for globular shape profiles was performed to estimate the R_g_, and additionally, using wider q range, the distance distribution function was estimated to estimate D_max_ and R_g_ (67). Considering the presence of flexible or loosely oriented segments, and domains in our protein shapes, we used chain-ensemble modeling protocol to restore shapes using the SAXS data and deduced parameters using GASBOR program (44). Earlier too, this methodology has been used to decipher domain-linker shapes (68, 69, 70). For GASBOR program, additional inputs were the number of dummy residues to be used for computing the shape, which were used equal to the dimeric state in case of applying P1 symmetry and equal to monomer when considering P2 symmetry. GASBOR jobs were run multiple times to obtain models, and all the models were analyzed. The MglB (crystal structure), MglC (crystal structure), and MglBC complex (docking models) structures were aligned with the models generated from GASBOR (44) using SUPCOMB (47). For the MglBC complex, the models obtained by docking were aligned with various GASBOR (44) generated models of the MglBC complex and then analyzed manually. The NSD for various GASBOR generated models obtained was calculated using damsel program of DAMAVER (71).

### Liposome cosedimentation assay

*E. coli* polar lipid extract was purchased from Avanti polar lipids. 1 mg/ml of the polar lipid extract was diluted with chloroform:methanol (1:3) and kept overnight at −20 °C. The mixture was then desiccated under a vacuum to create a thin layer of lipid. Liposomes were then prepared by resuspending a thin lipid film in 20-mM HEPES (pH 8.0) and 150 mM NaCl to a final concentration of 1 mg/ml. Liposomes were further extruded through a 0.4-μm polycarbonate filter (Avanti polar lipids). For cosedimentation assay, 10 μM of MglB, or MglC, or MglBC complex was mixed with 1 mg/ml of liposome and incubated for 30 min at room temperature. Samples were then ultracentrifuged at 45,000 r.p.m. for 1 h using the Beckman coulter SW 50.1 rotor. Control experiments were also performed without liposomes. Samples from supernatant and pellet were then visualized on SDS-PAGE after staining with Coomassie Brilliant Blue dye (Bio-Rad).

### Bioinformatics and structural analysis

PDBePISA (31) webserver was used for surface area calculations, and PDBsum (32) was used for obtaining protein subunit contact information. MglB and MglC were aligned together using SSM superpose in Coot (61). PDBeFold (39) was used for comparative analysis of MglC with other RLC7 family proteins. The number of residues aligned is given in Tables S1 and S2. PyMOL (The PyMOL Molecular Graphics System, Version 2.0, Schrödinger, LLC) was used to generate molecular graphic figures and to perform r.m.s.d. calculations. Electrostatic surface analysis was performed for using PyMOL Advanced Poisson-Boltzmann Solver plugin (38). For modeling of conserved residues on the MglC structure, the ConSurf server was used (33, 34, 35, 36, 37).

## Data availability

The atomic coordinates and structure factors for the reported crystal structure generated during this study are available at the Protein DataBank with accession code 7CT3 and 7CY1.

## Conflict of interest

The authors declare no conflict of interest in regard to this article.
