# Supplementary material for: Structural characterization of Myxococcus xanthus MglC, a component of the polarity control system, and its interactions with its paralog MglB
Source: J Biol Chem. 2021 Jan 22;296:100308. doi: 10.1016/j.jbc.2021.100308 (PMC7949163; doi:10.1016/j.jbc.2021.100308)
Supplement: Supplemental Figures S1–S10 and Tables S1–S4 [file mmc1.pdf]

Supplementary Information

Structural characterization of *Myxococcus xanthus* MglC, a component of polarity control system, and its interactions with MglB

Srajan Kapoor[1#], Akriti Kodesia[1#], Nidhi Kalidas[2], Ashish[2] and Krishan Gopal Thakur[1*]

[1]G. N. Ramachandran Protein Centre, Structural Biology Laboratory, Council of Scientific and Industrial Research-Institute of Microbial Technology, Chandigarh-160036, India

[2] G. N. Ramachandran Protein Centre, Council of Scientific and Industrial Research-Institute of Microbial Technology, Chandigarh-160036, India

#Equal contribution
*Correspondence
Krishan Gopal Thakur – Email: krishang@imtech.res.in

Table of Contents

Table S1. Comparative analysis of MglC monomer with other RLC7 family members

Table S2. Comparative analysis of MglC dimer with other RLC7 family members. The structural alignments and r.m.s.d. calculations were performed using PyMOL

Table S3. SAXS parameters for MglB, MglC, and MglB/MglC complex.

Table S4. Prediction of secondary structural content analysis of MglB and its mutants

Figure S1. Multiple Sequence alignment

Figure S2. MglC forms dimer

Figure S3. Comparison of MglC with other RLC7 family proteins

Figure S4. Analytical gel filtration profile suggests that MglB$^{\Delta CT}$ interacts with MglC

Figure S5. Surface analysis of probable MglC interacting face of MglB (PDB ID: 6HJM)

Figure S6. SAXS analysis of MglB$^{\Delta CT}$C

Figure S7. Low resolution solution structures of MglC, MglB and MglBC complexes fitted with different SAXS envelopes

Figure S8. Comparative analysis of CluPro generated models of MglBC complex with the SAXS data.

Figure S9. Interaction of MglC with MglB after site-directed mutagenesis.

Figure S10. Liposome Cosedimentation assay.

Table S1. Comparative analysis of MglC monomer with other RLC7 family members.

Protein	Organism	PDB ID : Chain	Z score	r.m.s.d. (Å)	%seq (Sequence identity)	%sse (% of matched secondary structure in target protein)	N_{align} (Number of residues aligned)	Angle of α2 helix compared to MglC α2 helix
LAMTOR2 - LAMTOR3 (Ragulator complex)	*H. sapiens*	5y3a : B	7.8	1.88	10	86	94	33.39
RLC7 domain	*S.avermitilis*	3kye : C	7.8	1.77	15	86	94	41.12
MglB MglA complex	*M. xanthus*	6h5b : C	8	1.77	9	86	94	41.29
MP1-P14 complex	*H. sapiens*, & *M. musculus*	1sko : B	7.4	2.01	10	86	94	33.64
MglB	*M. xanthus*	6hjm : D	7.7	1.91	9	86	93	37.63
MglB	*T.thermophilus*	3t1r : A	3.7	3.23	13	86	106	35.56
MP1-P14	N/A	2zl1 : B	7.1	2.14	9	86	93	33.65
LAMTOR2-LAMTOR3 (FLCN-FNIP-Rag-Ragulator complex)	*H. sapiens*	6ulg : B	2.9	3.3	7	57	101	30.79
Human regulator complex	*H. sapiens*	5yk3 : G	6.8	1.93	10	86	92	33.42
Raptor-Rag-Ragulator complex	*H. sapiens*	6u62 : E	3.7	3.19	5	86	98	27.31
Raptor-Rag-Ragulator complex	*H. sapiens*, & *M. musculus*	5x6v : G	5.8	2.25	11	71	96	31.58
Ragulator	*H. sapiens*	6b9x : B	3.6	3.06	5	86	95	35.56
Hepatitis B X-interacting protein	*H.sapiens*	3msh : A	6.2	2.17	11	57	73	34.21
Hepatitis B X-interacting protein	*H.sapiens*	3ms6 : A	5.9	2.29	11	71	75	36.07
MglA in complex with MglB in transition state	*T. thermophilus*	3t12 : C	3.9	3.19	13	86	106	31.45

Table S2. Comparative analysis of MglC dimer with other RLC7 family members. The structural alignments and r.m.s.d. calculations were performed using PyMOL.

Protein	Organism	PDB ID	r.m.s.d. (Å)	Number of residues aligned
LAMTOR2 -LAMTOR3 (Ragulator complex)	*H. sapiens*	5y3a	5.11	192
RLC7 domain	*S.avermitilis*	3kye	7.34	136
MglB MglA complex	*M. xanthus*	6h5b	5.89	176
MP1-p14 complex	*H. sapiens,& M. musculus*	1sko	4.54	208
MglB	*M. xanthus*	6hjm	5.29	192
MglB	*T. thermophilus*	3t1r	5.57	216
MP1-p14	N/A	2zl1	4.81	200
LAMTOR2-LAMTOR3 (FLCN-FNIP-Rag-Ragulator complex)	*H. sapiens*	6ulg	5.03	208
Human regulator complex	*H. sapiens*	5yk3	5.5	200
Raptor-Rag-Ragulator complex	*H. sapiens*	6u62	4.54	192
Raptor-Rag-Ragulator complex	*H. sapiens*, & *M. musculus*	5x6v	6.38	184
Ragulator	*H. sapiens*	6b9x	5.07	208
Hepatitis B X-interacting protein	*H.sapiens*	3msh	5.26	88
Hepatitis B X-interacting protein	*H.sapiens*	3ms6	4.28	120
MglA in complex with MglB in transition state	*T. thermophilus*	3t12	5.23	216

Table S3. SAXS parameters for MglB, MglC, and MglBC complex.

**Parameter**	**MglB**	**MglC**	**MglBC complex**	**MglB$^{\Delta CT}$MglC complex**
R_g (nm) (from Guinier)	3.35 ± 0.06	1.99 ± 0.03	3.69 ± 0.07	3.16 ± 0.13
R_g (nm) (from P(r))	3.51	2.10	4.07	3.24
I_0 (cm) (from Guinier)	135759 ± 1289.49	90162 ± 571.37	144938 ± 1509.42	375331.30 ± 884.75
I_0 (cm) (from P(r))	137600	91100	151800	37730
D_{max} (nm)	11.5	6.8	14.5	10.4
Porod volume (Å^3)	89,089	47,239	220,553	112,939
Molecular weight (theoretical) (kDa)	20.01	15.94	112.28	89.69
Molecular weight (observed) (kDa)	52.40	27.78	129.73	66.43
Shannon channel	8.849	8.891	13.19	8.358
Normalized special discrepancy (NSD)	1.586±0.051	0.855±0.019	1.646±0.137	1.319±0.047

Table S4. Prediction of secondary structural content analysis of MglB and its mutants

**Secondary structure**	**MglB**	**MglB^{S54A,S57A}**	**MglB^{E84A,R115A}**
α-helix (%)	18	18	18
β-sheet (%)	25.8	25.8	25.8
Turn (%)	13.7	13.7	13.7
Others (%)	42.4	42.4	42.4

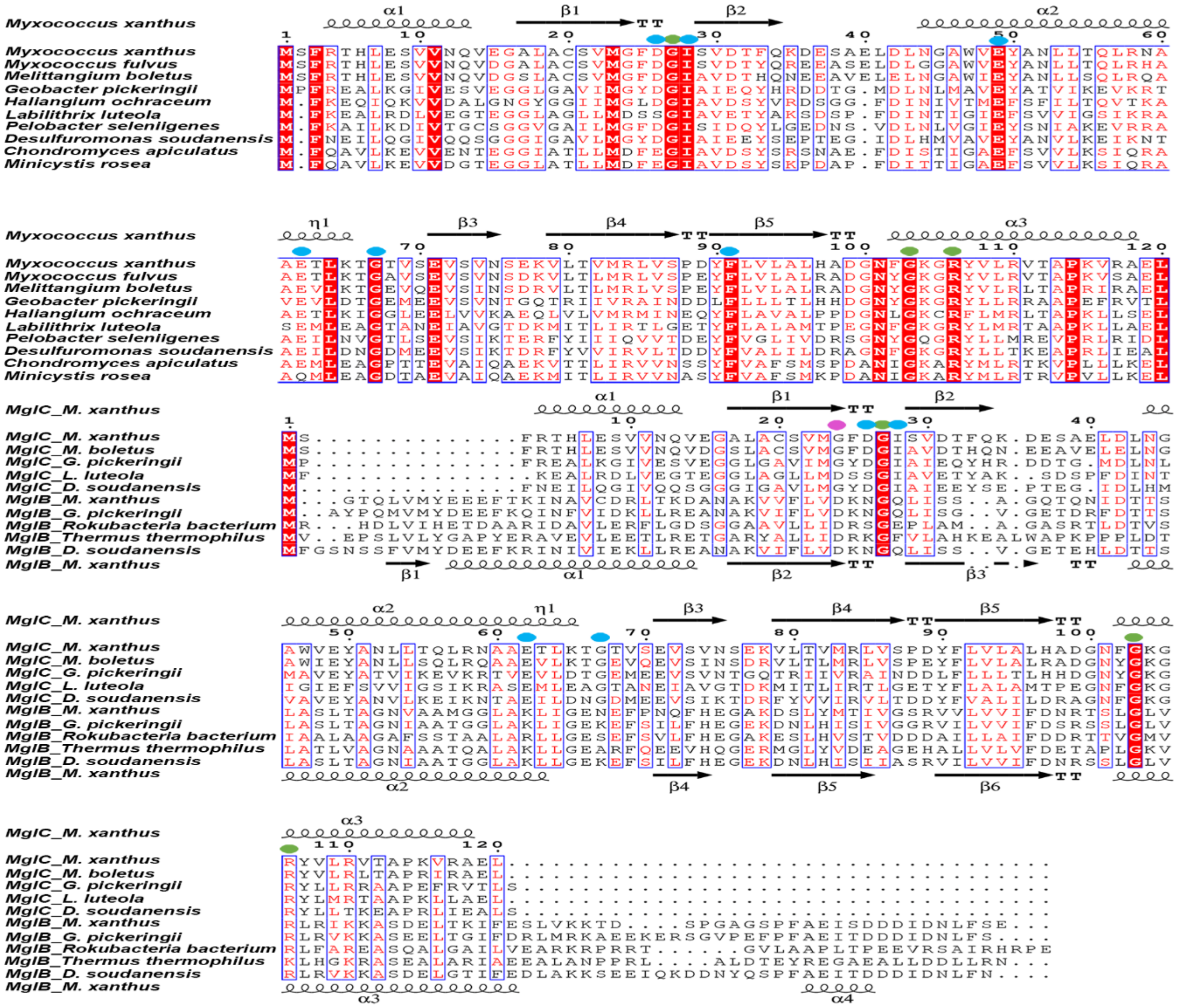

Figure S1. Multiple Sequence alignment

(A) Multiple Sequence Alignment of MglC homologs with more than 30% sequence identity **(B)** Multiple sequence alignment of MglC (more than 30% sequence identity) and MglB (more than 30% sequence identity) highlighting the conserved residues in MglB and MglC sequence by Green dots. The highly conserved residues in MglC that are mutated in MglB are marked by blue dots. The pink dot mark the residue conserved in MglB but mutated in MglC. (1-3).

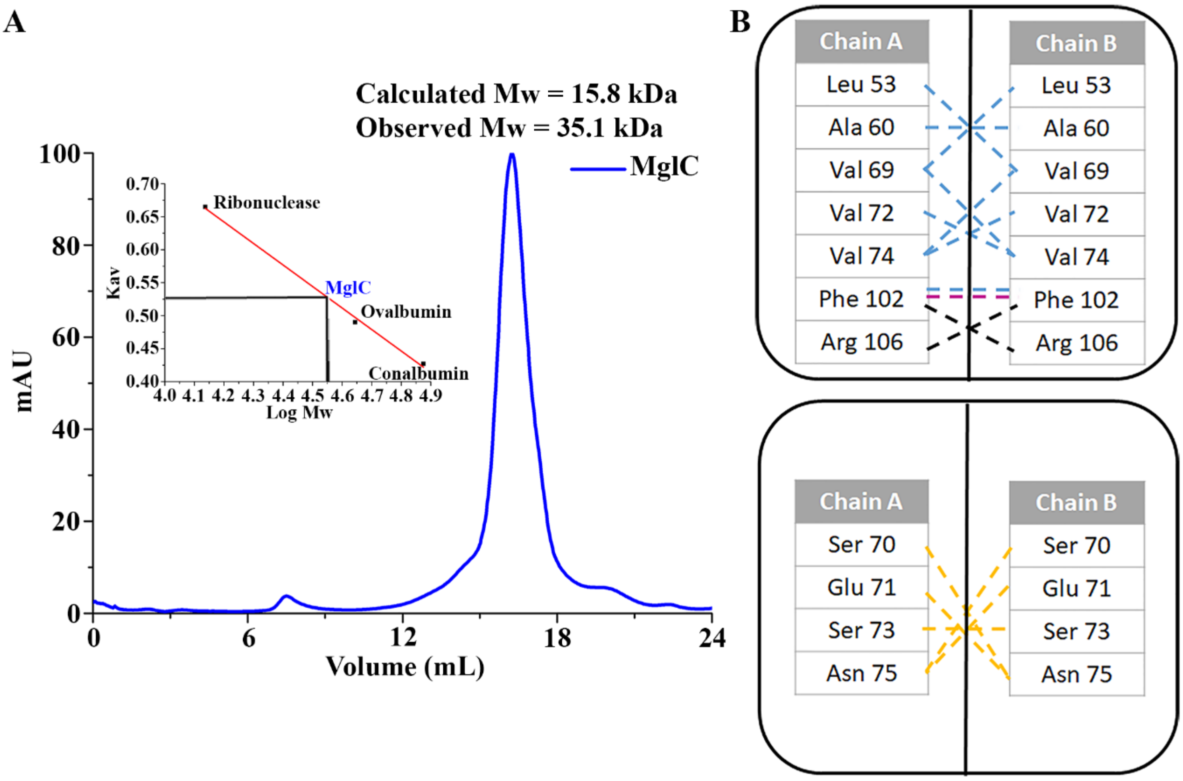

Figure S2. MglC forms dimer

(A) Gel filtration profile of MglC showing that MglC is obligate dimer **(B)** Structural analysis of MglC reveal the residues involved in interaction of dimer. Blue dotted lines represent hydrophobic contacts, yellow dotted lines represent hydrogen bonds, pink dotted lines represent aromatic-aromatic interactions and black dotted lines represent cation-pi interaction (4).

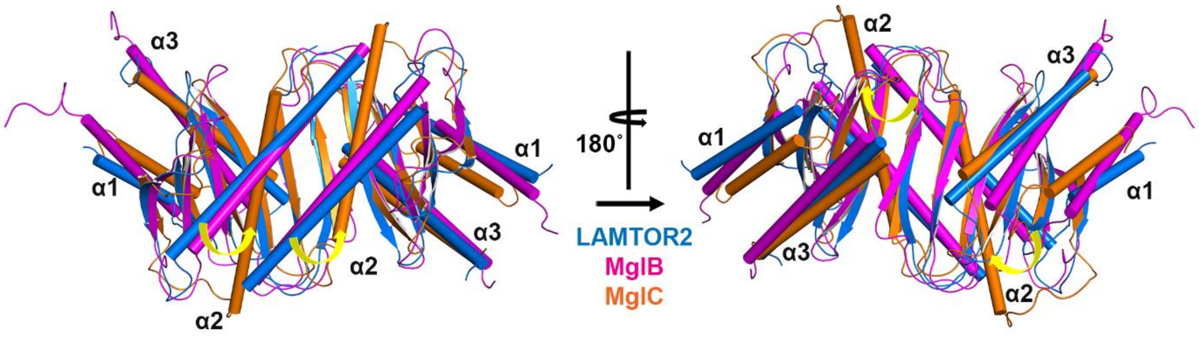

Figure S3. Comparison of MglC with other RLC7 family proteins.

Structural comparison of MglC dimer with the dimers of other RLC7 family proteins showing that in MglC dimer the α1 and α2 are also highly shifted compared to other RLC7 proteins.

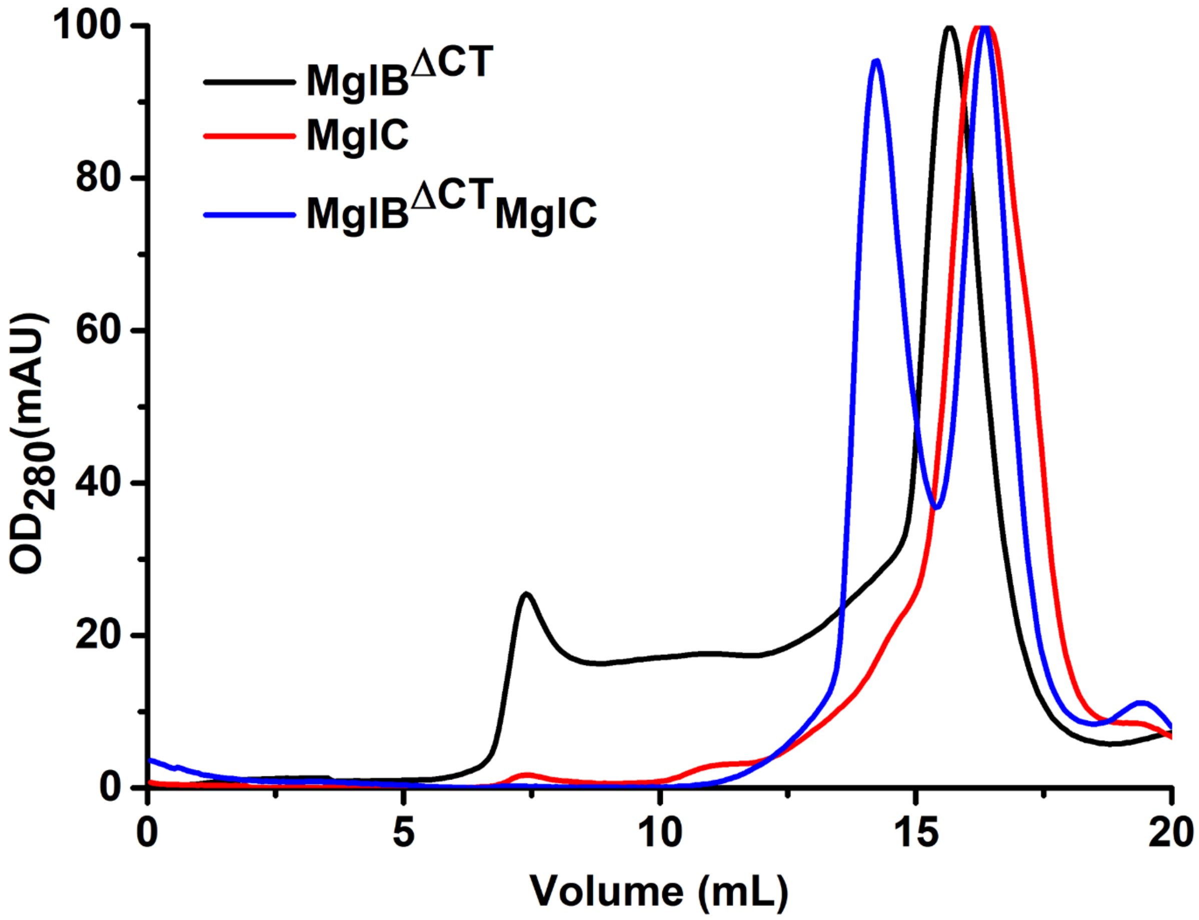

Figure S4. Analytical gel filtration profile suggests that MglB$^{\Delta CT}$ interacts with MglC.
(A) Purified MglB$^{\Delta CT}$ and MglC were mixed and run on the analytical gel filtration column. The gel filtration profile of TEV cleaved MglB$^{\Delta CT}$ (Black), TEV cleaved MglC (Red) and after mixing MglB$^{\Delta CT}$ and MglC (Blue) is shown. Blue line is having two peaks corresponding to MglB$^{\Delta CT}$-MglC complex and MglC. This indicates that MglB$^{\Delta CT}$ also interacts with MglC.

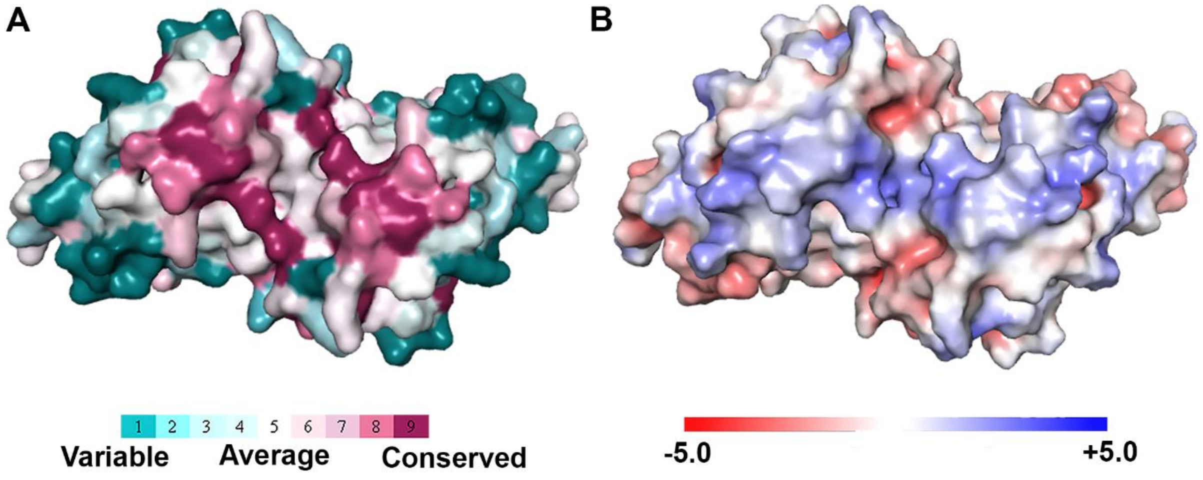

Figure S5. Surface analysis of probable MglC interacting face of MglB (PDB ID: 6HJM).
(A) ConSurf analysis of MglB **(B)** Electrostatic surface potential of MglB

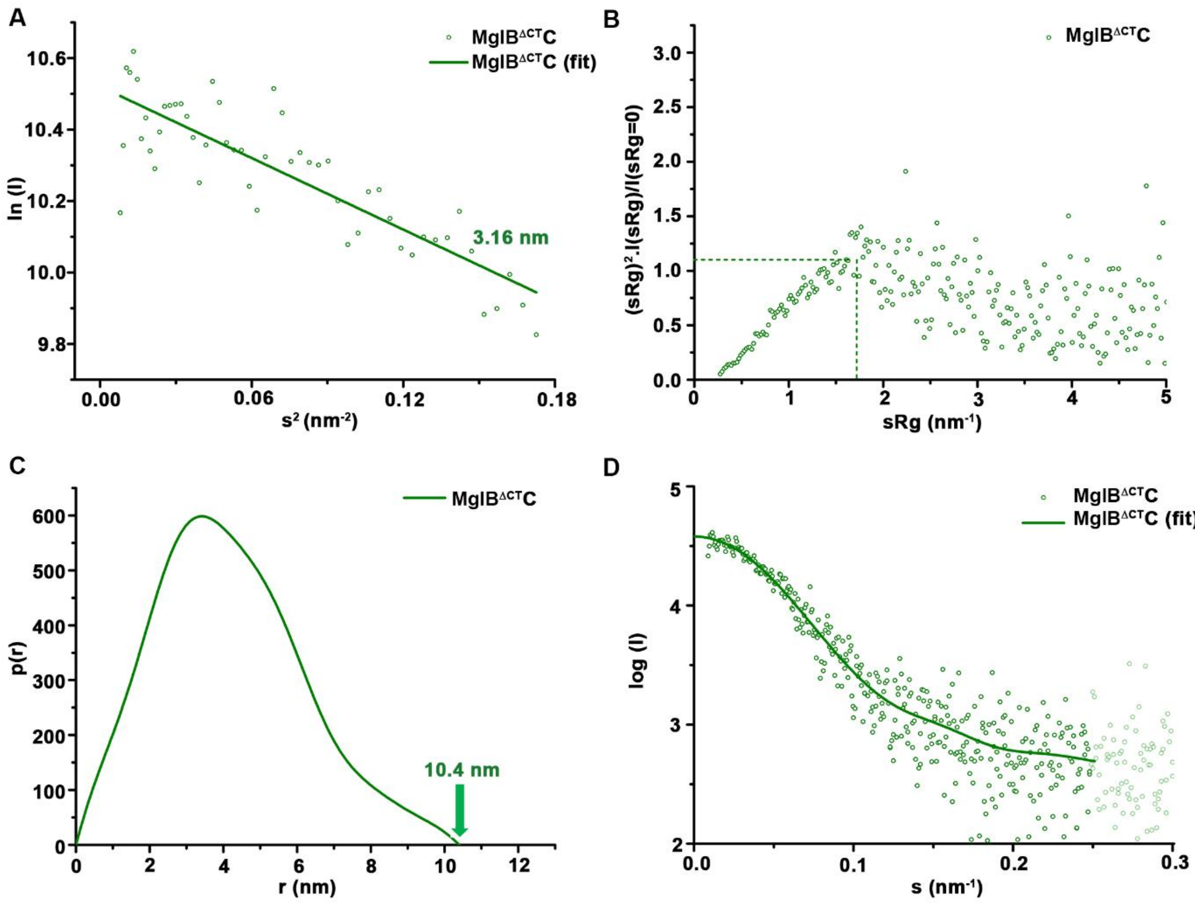

Figure S6. SAXS analysis of MglB$^{\Delta CT}$C.
(A) Guinier analysis of MglB$^{\Delta CT}$C (Rg = 3.16 nm) complex reveals linear fit with no signs of aggregation. **(B)** Dimensionless Kratky plot of MglB$^{\Delta CT}$C complex reveals that the complex is globular as the peak is located at the ideal position as shown by dotted lines. **(C)** Normalized pair distribution function P(r) analysis reveals the D_{max} of 10.4 nm. **(D)** Dummy atom model for MglB$^{\Delta CT}$C complex (χ^2 = 1.64) is prepared using GASBOR (5). The graph represents the intensity profile of MglB$^{\Delta CT}$C complex represent in spheres and the line represents the fitting of dummy atom model generated by GASBOR (5).

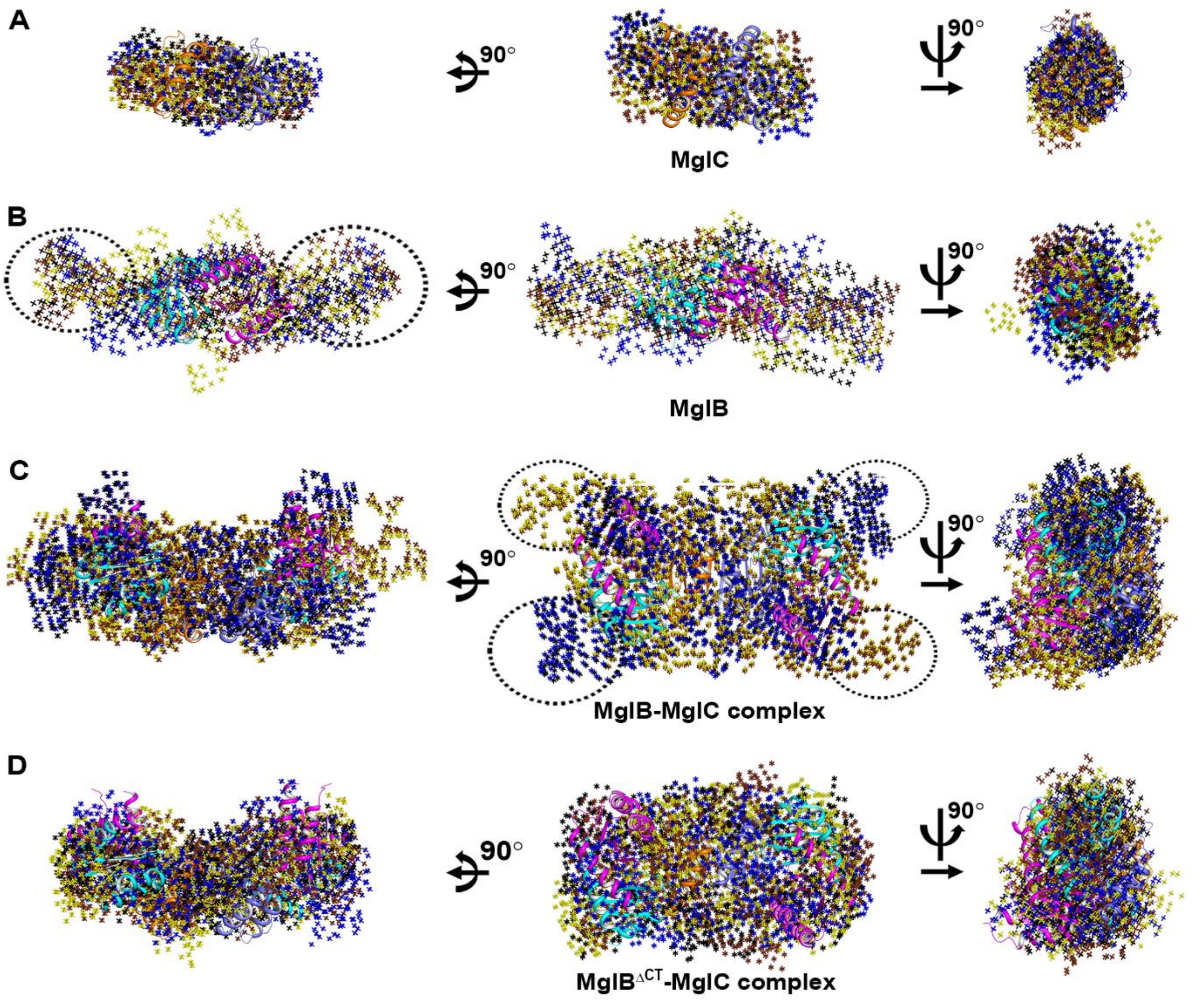

Figure S7. Low-resolution solution structures of MglC, MglB, and MglBC complexes fitted with different SAXS envelopes.

The region marked in the black circle corresponds to the region not resolved in the MglB crystal structure (PDB ID: 6HJM)

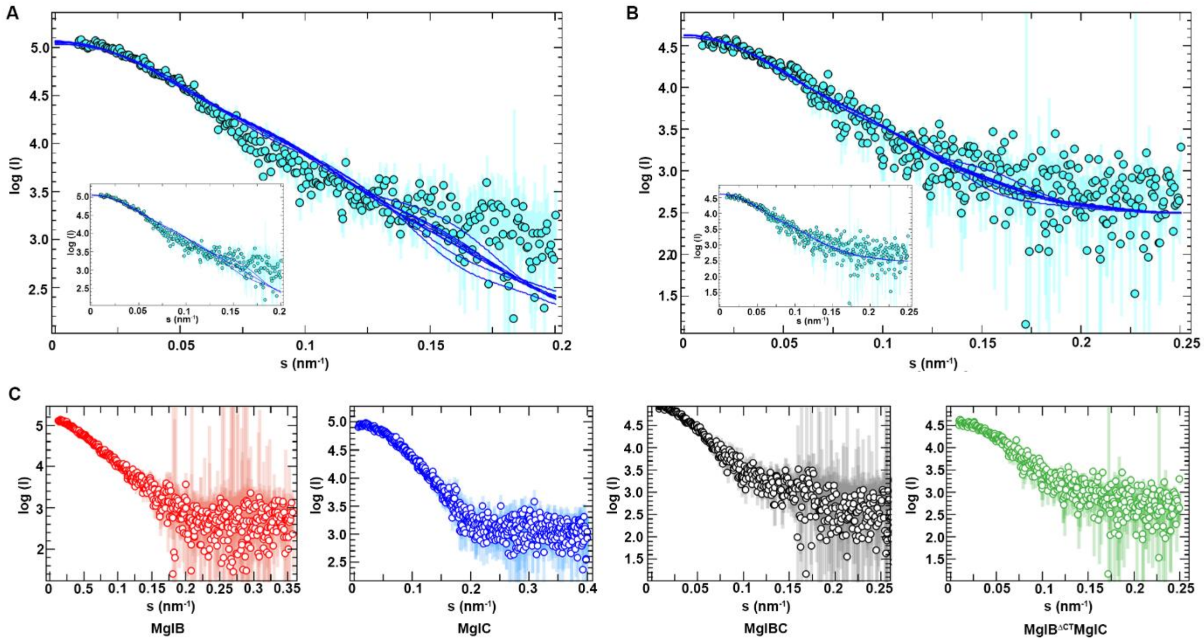

Figure S8. Comparative analysis of CluPro generated models of MglBC complex with the SAXS data.

(A) Fitting of MglBC models with SAXS data of MglBC complex with χ^2 of 2.109±0.16. **(B)** Fitting of MglBC models with SAXS data of MglB$^{\Delta CT}$C complex with χ^2 of 2.107±0.027. **(C)** log(I) vs s data of MglB, MglC, MglBC and MglB$^{\Delta CT}$C used for generationg GASBOR based dummy atom models.

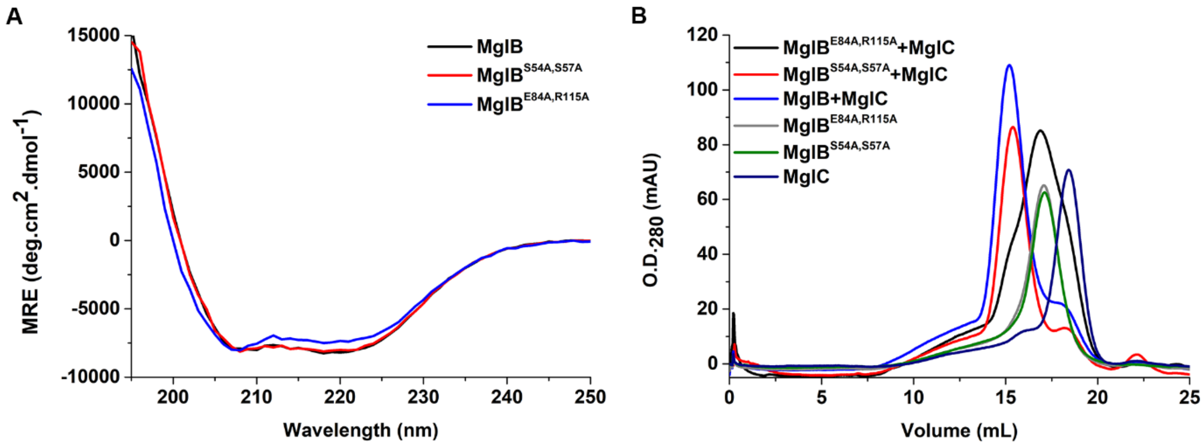

Figure S9. Interaction of MglC with MglB after site-directed mutagenesis.

(A) CD shows very little change in the secondary structural content of MglB upon site-directed mutagenesis. **(B)** MglB^{E84A,R115A} mutation leads to disruption of MglC/MglB interactions whereas, MglB^{S54AS57A} mutation did not cause any change in formation of MglC/MglB complex.

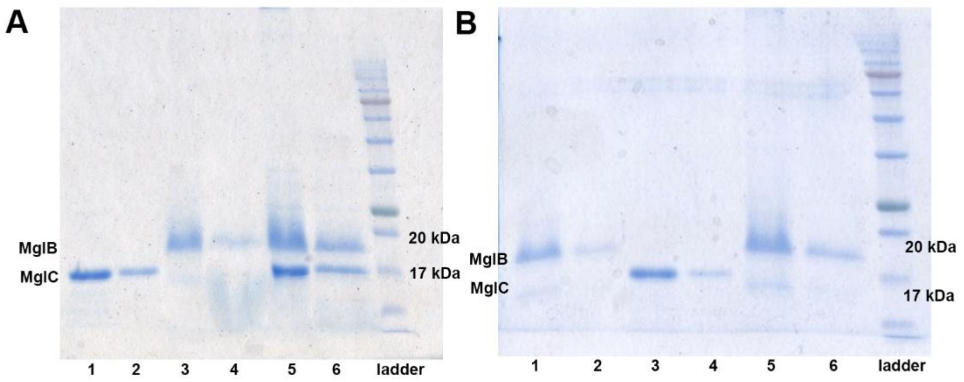

Figure S10. Liposome Cosedimentation assay.
(A) SDS-PAGE after ultracentrifugation of proteins incubated with liposomes. Lane 1- MglC pellet, Lane 2- MglC supernatant, Lane 3- MglB pellet, Lane 4- MglB supernatant, Lane 5- MglBC complex pellet, Lane 6- MglBC complex supernatant. **(B)** SDS-PAGE after ultracentrifugation of proteins as control**.** Lane 1- MglB pellet, Lane 2- MglB supernatant, Lane 3- MglC pellet, Lane 4- MglC supernatant, Lane 5- MglBC complex pellet, Lane 6- MglBC complex supernatant.
